# Molecular anatomy of the thalamic complex and the underlying transcription factors

**DOI:** 10.1007/s00429-015-1052-5

**Published:** 2015-05-12

**Authors:** Andrzej Nagalski, Luis Puelles, Michal Dabrowski, Tomasz Wegierski, Jacek Kuznicki, Marta B. Wisniewska

**Affiliations:** Laboratory of Neurodegeneration, International Institute of Molecular and Cell Biology, Warsaw, 02-109 Poland; Laboratory of Molecular Neurobiology, Centre of New Technologies, University of Warsaw, Warsaw, 00-927 Poland; Department of Human Anatomy, University of Murcia and IMIB, Murcia, 30071 Spain; Laboratory of Bioinformatics, Center of Neurobiology, Nencki Institute of Experimental Biology, Warsaw, 02-093 Poland

**Keywords:** Brain anatomy, Thalamus, Genoarchitecture, Transcription factors, TCF7L2

## Abstract

Thalamocortical loops have been implicated in the control of higher-order cognitive functions, but advances in our understanding of the molecular underpinnings of neocortical organization have not been accompanied by similar analyses in the thalamus. Using expression-based correlation maps and the manual mapping of mouse and human datasets available in the Allen Brain Atlas, we identified a few individual regions and several sets of molecularly related nuclei that partially overlap with the classic grouping that is based on topographical localization and thalamocortical connections. These new molecular divisions of the adult thalamic complex are defined by the combinatorial expression of *Tcf7l2*, *Lef1*, *Gbx2*, *Prox1*, *Pou4f1*, *Esrrg*, and *Six3* transcription factor genes. Further in silico and experimental analyses provided the evidence that TCF7L2 might be a pan-thalamic specifier. These results provide substantial insights into the “molecular logic” that underlies organization of the thalamic complex.

**Electronic supplementary material:**

The online version of this article (doi:10.1007/s00429-015-1052-5) contains supplementary material, which is available to authorized users.

## Introduction

The thalamus directly interacts with the cortex and is also a target for many other parts of the brain. Classically regarded as a simple relay station of sensory information, the thalamus actively regulates information transmission to the cortex (McAlonan et al. 2008; Bruno and Sakmann 2006; Saalmann and Kastner 2011; Xu and Südhof 2013; Constantinople and Bruno 2013), participates in motor control (Sommer and Wurtz 2006; Sommer 2003; Goldberg and Fee 2012), mediates communication between cortical areas, modulates cortico-cortical synchrony (Theyel et al. 2010; Purushothaman et al. 2012; Saalmann et al. 2012) and controls cortical states (Crunelli and Hughes 2010; Poulet et al. 2012). During development, the thalamus participates in cortical area patterning (Chou et al. 2013). Thus, understanding cognition and conscious awareness is not possible without detailed knowledge about the functioning of the thalamus.

The conventional thalamic complex can be divided into three diencephalic regions: prethalamus, thalamus (formerly known as the ventral thalamus and dorsal thalamus), and epithalamus (or habenula; Scholpp and Lumsden 2010; Martinez-Ferre and Martinez 2012; Puelles and Rubenstein 2003). Developmentally, the thalamus and epithalamus are derived from the alar plate of prosomere 2, and the prethalamus originates from the alar plate of prosomere 3 (Puelles and Rubenstein 2003; Puelles and Martinez 2013). In the early stages of development, these prosomeres are separated by the zona limitans intrathalamica (ZLI; Rendahl 1924), which is one of the key secondary organizers in the developing vertebrate brain that ensures properly patterned differentiation of the thalamus and prethalamus (Scholpp and Lumsden 2010). In adults, the thalamus proper can be further regionalized into distinct nuclei that are distinguishable from one another by differences in cell size and packing density, sources of specific afferents, and bidirectional connections with specific cortical regions. Classically, thalamic nuclei were categorized into several groups (i.e., periventricular or midline, medial, anterior, lateral, ventral, posterior, and intralaminar), primarily based on their topographical localization relative to the internal medullary lamina and precisely targeted termination of thalamocortical axons in the cortex (Puelles et al. 2012). The most distinctive and intensively studied thalamic nuclei are principal sensory nuclei, including the ventral posterolateral/ventral posteromedial (VPL/VPM), dorsal lateral geniculate (DLG), and medial geniculate nuclei (MGN) that project chiefly to individual somatosensory, visual, and auditory primary cortical areas, respectively. These represent key elements of our current knowledge of the organization and function of the thalamocortical system (Puelles et al. 2012). However, detailed mappings of thalamocortical connections have revealed that many thalamic nuclei target more than one neocortical area and may also send collaterals to subcortical or allocortical structures, such as the striatum, amygdala, hippocampus, and entorhinal cortex (Deschênes et al. 1998; Vertes 2006; Oh et al. 2014). Moreover, the thalamus has at least three subtypes of neurons, with differences in the spread of areal targeting, laminar pattern of arborization, and somatodendritic morphology (Clascá et al. 2012), in addition to some inhibitory interneurons. The unique properties (i.e., morphological and other) of given groups of neurons are conferred by molecular regulators of gene expression during differentiation and the mature state (Shirasaki and Pfaff 2002; Smidt and Burbach 2009; Hobert 2011; Baumgardt et al. 2007), thus the cytoarchitectonic and hodological picture should be complemented with molecular data to more deeply understand thalamic ontology and anatomy. Understanding how neurons are specified in the thalamus may be relevant to understanding the etiology of psychiatric conditions that are associated with abnormal thalamic functioning, such as schizophrenia (Pinault 2011; Cronenwett and Csernansky 2010; Byne et al. 2009; Parnaudeau et al. 2013).

Few analyses of differential gene expression in the thalamus have been performed, in contrast to other brain regions, such as the cerebral cortex (Belgard et al. 2011; Bernard et al. 2012; Molyneaux et al. 2007; Thompson et al. 2008; Siegert et al. 2012), hippocampus (Thompson et al. 2008), and retina (Siegert et al. 2012; Bassett and Wallace 2012). Recent studies have focused on the embryonic and early postnatal thalamus, providing a list of genes that might mediate the organization of thalamic nuclei, and many of these genes are dramatically downregulated during embryonic and postnatal development (Nakagawa and O’Leary 2001, 2003; Jones and Rubenstein 2004; Vue et al. 2007; Bluske et al. 2009; Yuge et al. 2011; Suzuki-Hirano et al. 2011). Nonetheless, we still have very little knowledge about the molecular determinants of thalamic neuronal identities in the adult brain.

The aim of the present study was to reexamine the relationship between cytoarchitectonically delineated thalamic nuclei in the mouse using gene expression data that are available in the Allen Brain Atlas database (Ng et al. 2009; Lein et al. 2007) and identify key transcription factors that underlie specific gene expression patterns in different parts of the thalamic complex.

## Results

**Fig. 1:**
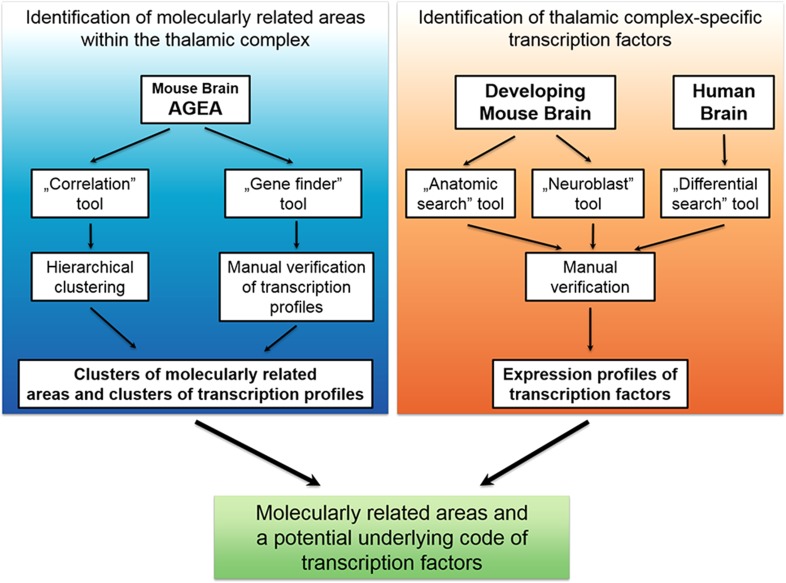
Schematic diagram summarizing critical steps of the analysis

**Fig. 2:**
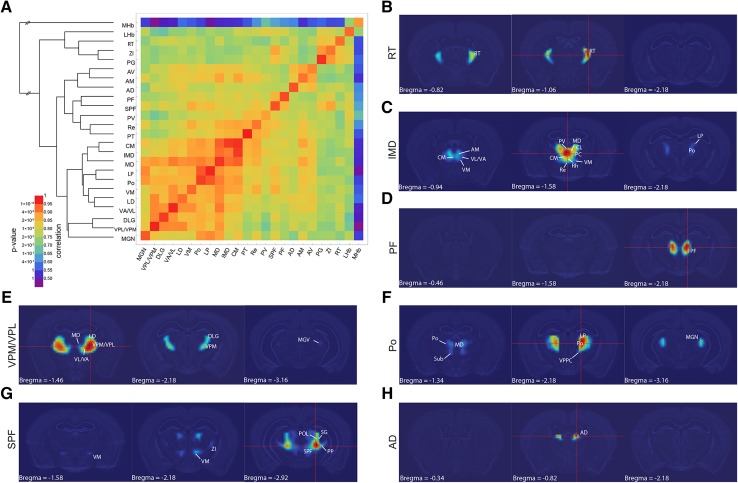
Correlation of gene expression between nuclei of the thalamic complex. **a** Matrix of correlation voxels taken from the AGEA atlas for fixed points that were placed in individual nuclei of the thalamic complex. The *color scale* represents the mean Pearson correlations that were calculated between each pair of nuclei, together with the corresponding *P* values. These values were clustered using the hierarchical cluster analysis method. All of the correlation data are available in Supplementary Material 1, including means and variances. **b**–**h** AGEA correlation maps for seed voxels placed in **b** RT, **c** IMD, **d** PF, **e** VPM/VPL, **f** Po, **g** SPF, and **h** AD. The computed correlation values are displayed with false-color images using a blue-to-red color scale (“heat map”), with the threshold interval set to (0.9, 1). For a detailed description of AGEA spatial gene expression correlation maps, see Ng et al. (2009). Three panels, ordered from rostral to caudal, are shown for each seed voxel

**Fig. 3:**
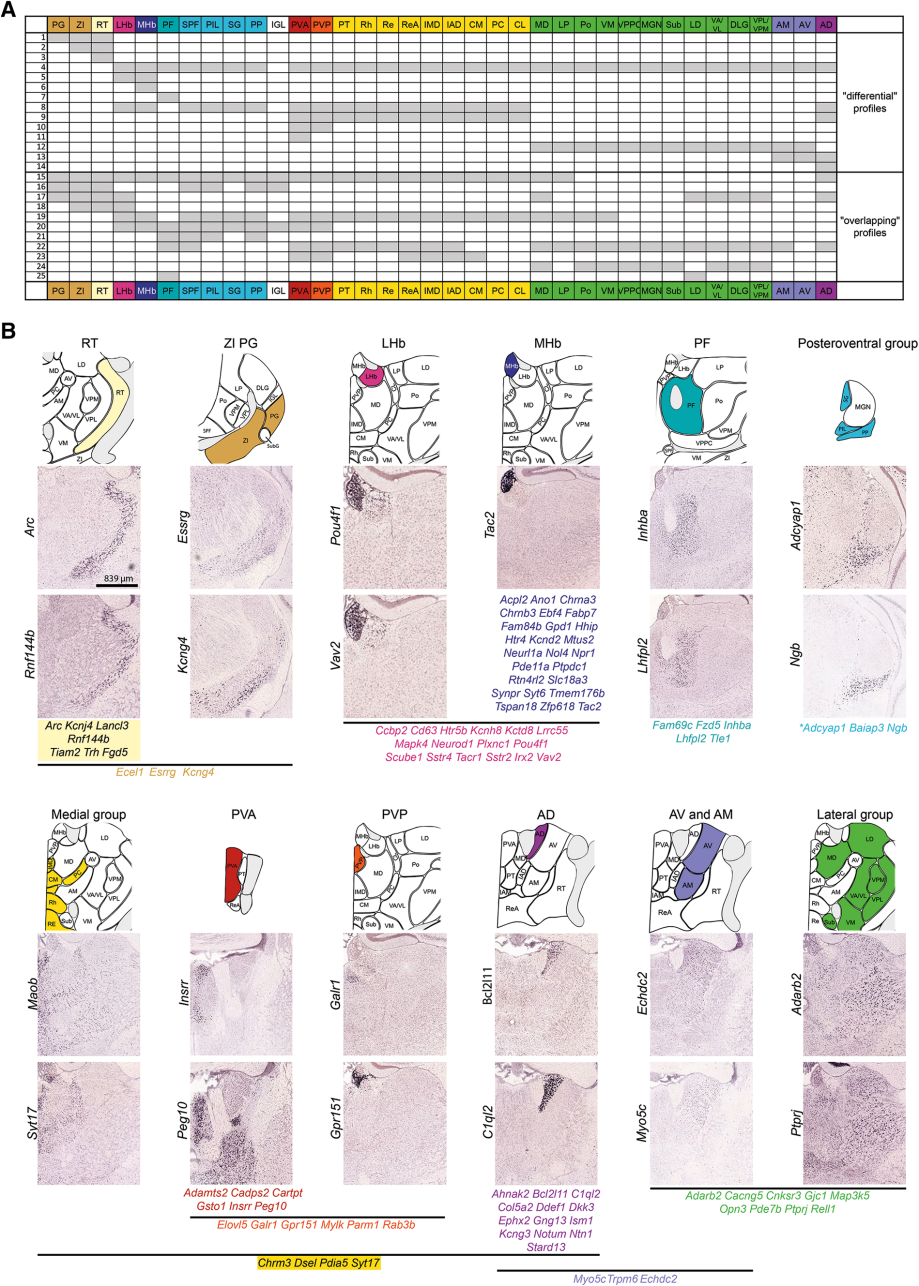
Expression of specific genes in different areas of the thalamic complex. **a** Expression profile matrix of thalamic complex nuclei. **b** In situ hybridization data for two selected mRNAs that exemplify gene expression boundaries in the thalamic complex and lists of some other genes with similar patterns of expression. Digital images of representative gene expression were downloaded from the ABA gene expression atlas. All of the images have the same magnification. The *scale bar* is displayed in the *lower left corner* of the *Arc* image

The Anatomic Gene Expression Atlas (AGEA) is a gene expression-derived atlas that is based on an in situ hybridization dataset of over 4000 genes (Ng et al. 2009). The expression data are mapped to a common coordinated anatomical framework that allows comparison of the degree of gene expression profile similarity (via Pearson’s correlation) between a user-specified seed voxel and other brain areas. We used this tool to compare transcriptional profiles between various nuclei of the adult thalamus, epithalamus, and prethalamus (Fig. 1). We collected mean Pearson’s correlations within conventional anatomical delineations for each of 24 nuclei of the thalamic complex relative to every other nucleus. All correlation scores and means are provided in Supplementary Material 1. The higher the correlation value between two nuclei, indicated by the heat-map, the more genes from the input set are co-expressed and the fewer genes are differentially expressed. To determine the molecular relationships between nuclei, the correlation values were clustered using the hierarchical cluster analysis method (Fig. 2a). All analyzed nuclei were also ranked by increasing intrastructural correlation to show their homogeneity level (Fig. S1a). Next, to determine exactly which genes delineate these groups, we used the AGEA Gene Finder tool (Ng et al. 2009). The resulting lists were manually inspected to reject data that originated from staining artifacts. The anterior/posterior parts of the PVP and Re nuclei (PVA/PVP and Re/ReA, respectively) were analyzed separately. Additionally, nine small nuclei that were not analyzed using the seed-voxel method were included in this analysis. Spatial expression was individually analyzed for the resulting 230 genes. A dozen repeated expression patterns were observed for these genes (Supplementary Material 2), which allowed grouping the nuclei into clusters (Fig. 3a, b).

Both methods (i.e., the hierarchical clustering of mean Pearson correlations and the comparison of expression profiles) divided the thalamic complex into the thalamus proper, the prethalamus, and two outliers: the LHb and MHb of the epithalamus, of which the LHb showed weak similarity to the prethalamus. The examination of molecular similarity between the nuclei of the thalamus proper allowed dividing it into several correlated groups and revealed a few outliers. A detailed partition of the thalamic complex into main groups and subgroups, together with the underlying genes, is described below.

### Prethalamus

Within the group of prethalamic nuclei, we observed some correlation in gene expression between the ZI and PG (former ventral lateral geniculate nucleus) and SPF of the thalamus proper, whereas the correlations between RT and ZI and between RT and PG were quite low (top part of the dendrogram in Fig. 2a, b). Comparisons of the expression profiles suggested a closer relationship between ZI, PG, and RT but revealed seven genes that were expressed exclusively in the RT (Supplementary Material 2, profiles 1–3; Fig. 3a, b). Within the RT and ZI, relatively low intrastructural correlation values were observed, which suggest gene expression heterogeneity (Fig. S1a, b and c). Between the genes which were expressed in all prethalamic nuclei (Supplementary Material 2) there were genes associated with the phenotype of γ-aminobutyric acid (GABA)-ergic neurons: *Gad1*, *Gad2*, and *Slc32a1* (vesicular GABA transporter). These genes were also expressed in the SPF, PIL, and PP of the thalamus proper and the IGL (Supplementary Material 2, profile 16; Fig. 3a). Many genes that were expressed in the prethalamus were also expressed in the LHb (Supplementary Material 2, profiles 17 and 18; Fig. 3a).

### MHb and LHb

The molecular profile of the MHb appeared to be entirely different from the rest of the thalamic complex, with the exception of a weak relationship with the neighboring LHb (top part of the dendrogram in Fig. 2a). Ten genes were expressed only in the MHb and LHb (Supplementary Material 2, profile 5; Fig. 3a, b), and many genes that were expressed in the MHb and LHb were also expressed in the SPF, PIL, SG, PP, PV, and midline and intralaminar thalamic nuclei (profiles 8, 19 and 20). Nevertheless, as many as 23 genes were expressed exclusively in the MHb (profile 6; Fig. 3b). This feature makes the MHb an outlier within the thalamic complex. Consequently, the LHb was more correlated with the prethalamic nuclei and some parts of the thalamus proper than with the MHb (profiles 17 and 18; top part of the dendrogram in Fig. 2a). Both epithalamic nuclei, and especially MHb, showed low intrastructural correlation, indicating that they are heterogenous in their gene expression profile (Fig. S1a).

### Thalamus

The seed voxel-based examination of gene expression similarity and the cluster analysis revealed several coherent and overlapping areas in the thalamus (Fig. 2a). This grouping was further refined by common expression profiles (Supplementary Material 2; Fig. 3a).

The first assembly consisted of (1) classic midline nuclei: PT, Re [further divided into the Re and anterior Re (ReA) based on their relatively low intrastructural correlation values; Fig. S1a, e], Rh, and IMD; (2) rostral intralaminar nuclei: CM, PC, and CL; and (3) median anterior nuclei: IAD and IAM (Fig. 2a, c and S2a, b). We called this group “medial” (Supplementary Material 2, profile 9; Fig. 3a, b). The group shared many genes with the SPF, PIL, SG, and PP as well as with the epithalamus and AD (profiles 8, 9 and 19).

The PV, further divided into posterior part (PVP) and anterior part (PVA) because of its relatively low intrastructural correlation values (Fig. S1a, d), appeared to cluster with the medial group, but it differed from this group by expressing a dozen genes that were specific to this region (Supplementary Material 2, profiles 9–11; Fig. 3a). The PVA/PVP also expressed a number of genes together with the SPF, PIL, SG, PP, IGL and PF, which did not show expression in the medial cluster (profile 20).

The PF, a supposedly caudal intralaminar nucleus (Van der Werf et al. 2002), unlike the other intralaminar nuclei, did not cluster with the medial group. The PF exhibited a unique gene expression pattern that was marked by five genes with restricted expression (Supplementary Material 2, profile 7; Fig. 2a, d; Fig. 3a, b).

Small caudally located nuclei (SPF, PP, PIL, and SG) constituted the second assembly, which we named “posteroventral” (Fig. 2g, Fig. 3b). The IGL appeared to share molecular characteristics with this group, particularly with the PP. Forty-two of 47 genes that were expressed in the IGL were also expressed in the PP (Supplementary Material 2). Gene expression in the posteroventral group partially overlapped with the profile of the prethalamus and epithalamus, PF, PVA/PVP, and medial cluster (profiles 8 and 15). Half of the genes that were expressed in this group were also expressed in pretectal and hypothalamic nuclei (data not shown).

The third group comprised the ventral nuclei (i.e., VPM/VPL, VA/VL, VM, VPPC, and DLG; Fig. 2e), MGN [which could be further divided into ventral (MGV), dorsal (MGD), and medial (MGM) parts; Fig. S1F], Sub (Fig. S2c), LD, LP, Po, and MD nuclei (Fig. 2f). We termed this group “lateral” (Supplementary Material 2, profile 12; Fig. 3a, b). This cluster also expressed a set of genes that were expressed in the AM and AV (profile 12), in the posteroventral and medial clusters, PVP/PVA, and epithalamus (profile 4). Nevertheless, the nuclei that comprised the lateral group did not constitute a homogeneous assembly (profiles 15, 17, 19, 22 and 24). For example, the VPM/VPL, VA/VL, LD, DLG, and MD were distinguished by the expression of genes that were characteristic also for the AD, prethalamus, and LHb (profile 17). Among nuclei in this group, DLG, MD, LP, VA/VL, VPL/VPM showed relatively high intrastructural correlation values (Fig. S1A), suggesting homogeneity in their gene expression patterns, whereas intrastructural correlation values for LD, VM and MGN were lower.

The classic anterior nuclei (AM, AV, and AD) clustered into one group (Fig. 2a), delineated by the specific expression of few genes (Supplementary Material 2, profile 13, Fig. 3a, b). However, the AD apparently differed from the AM and AV (Fig. 2a, h, S3e, f). As many as 10 genes were expressed exclusively in the AD (profile 14). Gene expression profiles linked the AM and AV to the lateral group (profiles 12 and 22), but many genes that were expressed in this group were not expressed in the AD or/and AM and AV (profiles 12, 22 and 24). AM and AV showed relatively low intrastructural correlation (Fig. S1a), which was not further analyzed.

### Transcription factor code for the adult thalamic complex

**Fig. 4:**
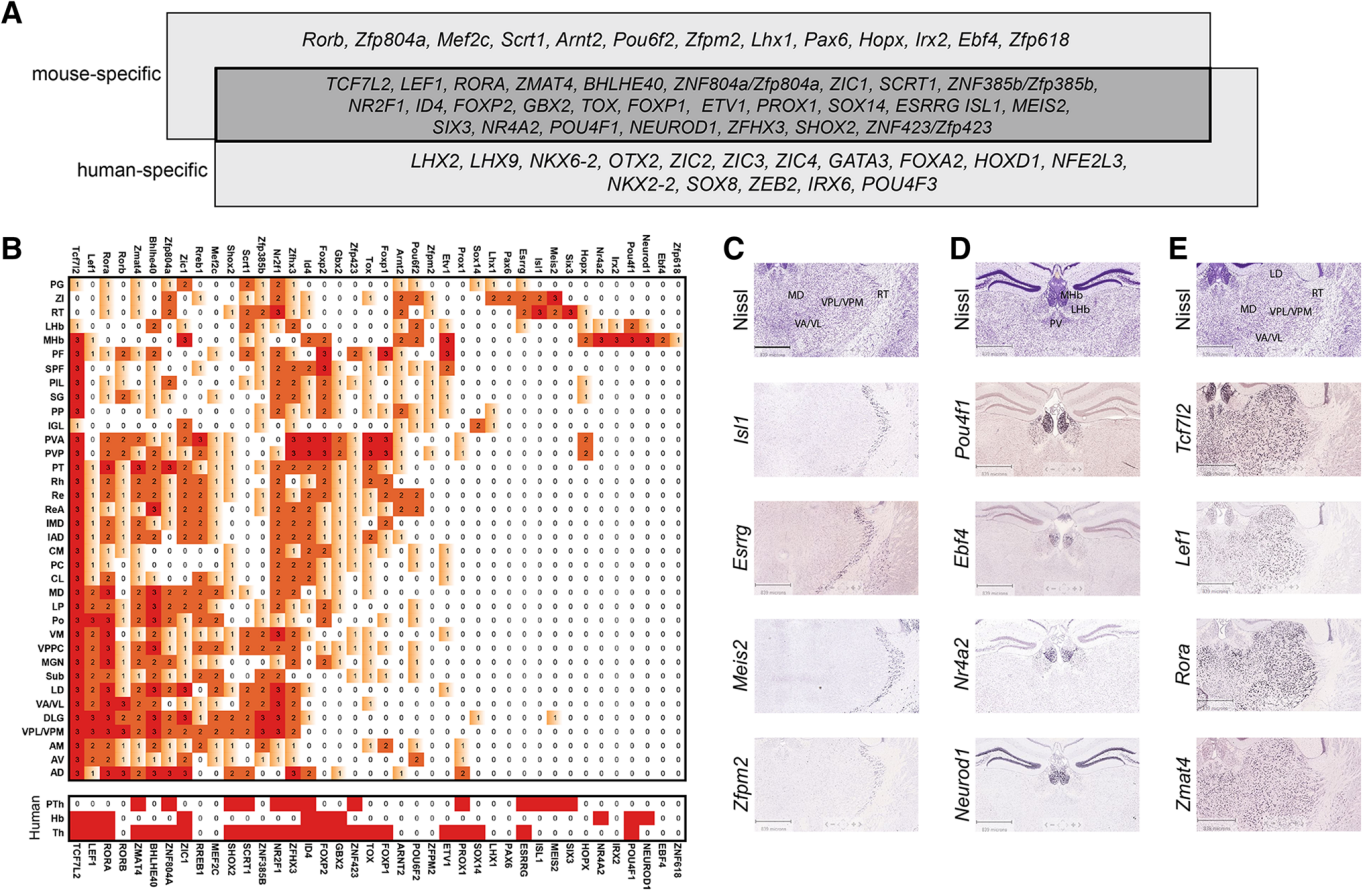
Transcription factors differentially expressed in nuclei of the thalamic complex. **a** Species-specific expression of transcription factor genes in the human and mouse thalamic complex. **b** The *upper panel* summarizes the relative expression intensity of the listed mouse transcription factor genes, which was estimated as high (3), medium (2), or low level/scattered (1) expression or undetected (0). The *lower panel* summarizes the expression profiles of human orthologs of the listed mouse genes for transcription factors in prethalamus (PTh), habenulae (Hb) and thalamus (Th). The expression of a gene is indicated in *red*. **c**–**e** Example ISH images of transcription factor genes that were expressed in **c** prethalamic, **d** epithalamic, and **e** thalamic nuclei. All of the digital images were downloaded from the ABA gene expression atlas. *Scale bar* in *C* = 839 μm

Transcription factors are key regulators of neuronal cell fate specification during development, and they maintain the molecular identity of neurons in the adult. To identify transcription factors that are specifically expressed in the adult thalamic complex and compare their expression patterns with the identified molecular domains, we used the Anatomic Search and Neuroblast tools that are available in the Developing Mouse Brain Atlas (http://developingmouse.brain-map.org; accessed July 30, 2014), as well as Differential Search tool from Human Brain Atlas (http://human.brain-map.org/, accessed February 23, 2015) (Fig. 1). We identified 56 genes for transcription factors that showed clearly restricted expression in some areas of the adult mouse and human thalamic complex (Fig. 4a). 27 of these genes were expressed in both species. Next, for each of the mouse genes, we estimated the intensity of the in situ hybridization (ISH) signal in every nucleus of the thalamic complex (Fig. 4b). Some of these genes were broadly expressed, whereas others were expressed in more restricted areas or in individual nuclei. Because there are no in situ data for all transcription factors in human brain, we only evaluated expression in the prethalamus, thalamus and epithalamus (Fig. 4b bottom panel). The data interpretation was focused on the mouse thalamic complex unless stated otherwise.

We did not find any specific marker of the whole adult prethalamus, although *Six3* showed expression in the RT, *Pax6* showed expression in the ZI, and *Meis2* and *Isl1* showed expression in both nuclei (Fig. 4c). All these genes for transcription factors, except *Pax6*, were also expressed in human prethalamus. Many transcription factors (e.g., *Rora*, *Zmat4*, *Nr2f1*, *Zfpm2*, and *Essrg*) were expressed in at least one prethalamic nucleus and several thalamic and/or epithalamic nuclei. In contrast, we readily found several epithalamus-restricted transcription factors (Fig. 4d) and factors that were specific for thalamic groups (Fig. 4e). *Pou4f1* (*Brn3a*) was expressed in the entire epithalamus. *Irx2*, *Nr4a2*, and *Neurod1* showed expression in a subset of MHb and LHb cells. *Zfp618* and *Ebf4* were expressed in some parts of the MHb. *Pou4f1, Nr4a2*, and *Neurod1* were also specific for the mouse epithalamus. Both thalamic and epithalamic derivatives of the alar part of prosomere 2 in mouse and human were clearly delineated by the expression of *Tcf7l2*, although only a subset of cells were marked within the LHb in mouse. *Lef1* showed especially strong expression in the lateral group and AM/AV and lower expression in the medial group, PF, AD, and MHb. *Gbx2* was expressed in the medial and posteroventral groups and only in some nuclei from the lateral group (MP, LP, MGN, and VPPC). *Id4* strongly marked medial and posteroventral nuclei and the AD. All of the anterior nuclei were marked by *Prox1* expression, which was also present in the PVP/PVA, whereas the AD lacked *Nr2f1* expression, which was otherwise expressed everywhere in the thalamic complex, with the exception of the AD, MHb, PVA, and IGL. The PF, especially its lateral part, was marked by high *Etv1* expression, which was also present in the posteroventral group, intralaminar nuclei (CM, PC, and CL), and ventral part of the MHb. *Gbx2* and *Id4*, which were specific for the medial and posteroventral nuclei, were absent from the PF. The IGL expressed only few transcription factors, including *Lhx1*, which in the thalamus was expressed only in the PP.

In conclusion, the spatial expression of transcription factors was generally consistent with the patterns of correlated gene expression in the thalamic complex. Seemingly, however, the molecular identity of diverse thalamic areas might be determined by combinations of transcription factors, each of which can be broadly expressed, rather than by single specific factors. We also observed that expression profiles of genes for transcription factors are strongly conserved between mouse and human.

### Genes with LEF/TCF binding motifs are overrepresented among thalamus-specific genes

We sought to further determine whether the group of genes whose expression is enriched in the thalamus and epithalamus exhibits signatures that are indicative of regulation by TCF7L2, which delineates prosomer 2, and the related transcription factor LEF1 that is present in most of prosomer 2 area. We first used a bioinformatic approach to identify potential LEF/TCF binding sites within conserved regulatory regions of genes that are enriched in the thalamus and epithalamus, and analyze their representation compared with the motif occurrence in all 4,206 genes (i.e., a background list) from the Allen Mouse Brain Atlas database (see Supplementary Material 3 for gene lists). We found significant overrepresentation of genes with LEF1/TCF motifs (highest *p*_val_ = 0.04, two-tailed Fisher’s exact test, followed by Bonferroni correction for multiple comparisons). The same analysis, but limited to transcription factor genes (*n* = 38) compared against the background of all transcription factor genes from the Allen Mouse Brain Atlas database (681 genes; see Supplementary Material 4 for gene lists), yielded a similar result (highest corrected *p*_val_ < 0.02). These results suggest that TCF7L2 and LEF1 are strong candidates for master regulators of genes that are specific to the thalamus and epithalamus, including genes that encode area-specific transcription factors.

### TCF7L2 and LEF1 are genuine regulators of thalamus-specific transcription factors

**Fig. 5:**
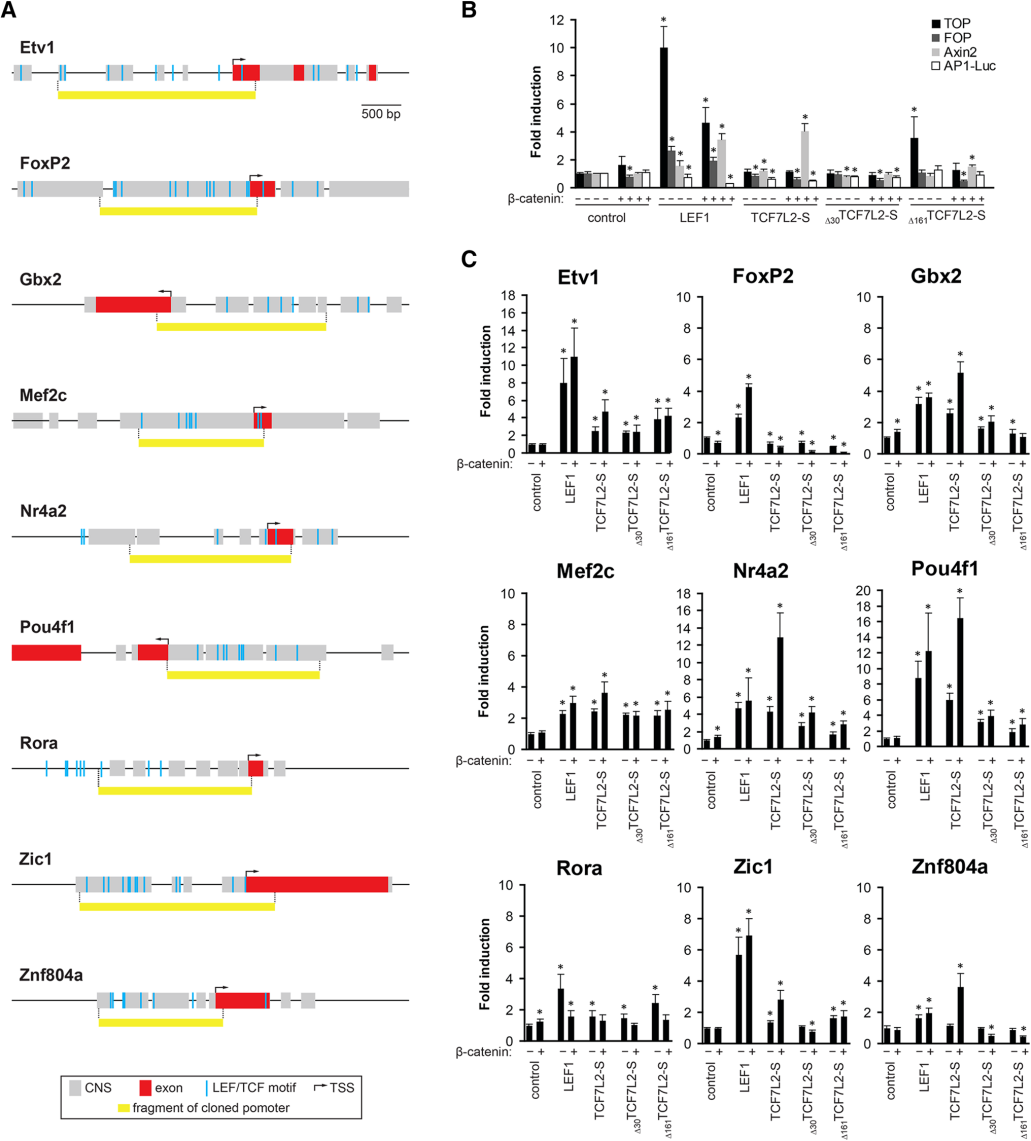
Thalamus- and epithalamus-specific transcription factors are activated by LEF1 and TCF7L2-S. **a** Schematic representation of the regulatory regions of the *Etv1*, *Foxp2*, *Gbx2*, *Mef2c*, *Nr4a2*, *Pou4f1*, *Rora*, *Zic1*, and *Znf804a* genes. Human-to-mouse conserved non-coding sequences are represented as *gray boxes*, and exons are shown in *red*. The positions of the LEF1/TCF motifs are marked as *blue bars*. *Yellow boxes* indicate the cloned parts of the promoters that were placed upstream of the luciferase reporter. Transcription start sites and the direction of transcription are indicated by *arrows*. **b** Activation of LEF1/TCF reporters TOP and Axin2-Luc and negative controls FOP and AP1-Luc by LEF1, TCF7L2-S, _Δ30_TCF7L2-S, and _Δ161_TCF7L2-S, with or without β-catenin, in Hela cells. The data are expressed as mean ± SD (*n* = 2–4 duplicated experiments). Mean values of luciferase activity (relative light units) obtained for a reporter alone were set to 1, and other values were related accordingly. Renilla luciferase reporter plasmid was used to normalize the results. Significant differences relative to the control are indicated with an *asterisk* (*P* value <0.5, two-tailed Mann–Whitney test). **c** Activation of the investigated promoters (depicted in **a**) by LEF1, TCF7L2-S, _Δ30_TCF7L2-S, and _Δ161_TCF7L2-S, with or without β-catenin, in Hela cells. Mean values obtained for a promoter alone (control) were set as 1, and other values were related accordingly. Renilla luciferase reporter plasmid was used to normalize the results. The data are expressed as mean ± SD (*n* = 2–4 independent experiments, each with duplicate samples). *Asterisks* indicate significant difference relative to the promoter alone (*P* value <0.5 calculated with two-tailed Mann–Whitney test)

To test the above hypothesis, we cloned the promoters of nine thalamic/epithalamic transcription factor genes: *Etv1*, *Foxp2*, *Gbx2*, *Mef2c*, *Nr4a2*, *Pou4f1*, *Rora*, *Zic1*, and *Znf804a*. The cloned fragments, approximately 2.5 kb long, encompassed the conserved putative promoter regions and contained clusters of LEF1/TCF motifs (Fig. 5a). We linked them with a luciferase reporter and assayed their activity in HeLa cells in the presence of TCF7L2 or LEF1, with or without β-catenin. The HeLa cell line was selected because of low level of the endogenous TCF7L2 protein (Frietze et al. 2012; Nagalski et al. 2013). Our previous work showed that thalamic neurons express specific isoforms of TCF7L2 (TCF7L2-S isoform and dominant-negative _Δ161_TCF7L2-S isoform, the latter predominating in the embryonic thalamus) and LEF1 (Nagalski et al. 2013). Therefore, we focused on the effects of LEF1, TCF7L2-S, and _Δ161_TCF7L2-S on the activity of the promoters. We also used _Δ30_TCF7L2-S, a dominant-negative form that lacks only the β-catenin-binding domain.

As positive controls or references, we used Super8xTOPflash (TOP) with multimeric LEF1/TCF binding sites and the *Axin2* promoter (Axin-Luc; Fig. 5b). Super8xFOPflash (FOP; with LEF1/TCF binding sites mutated) and AP1 reporter (AP1-Luc) served as negative controls. LEF1 potently activated TOP and, to a lesser extent, the *Axin2* promoter when expressed alone or with β-catenin.As expected, LEF1 weakly activated FOP and did not activate AP1-Luc. TCF7L2-S alone did not activate any control promoters, but it activated the *Axin2* promoter when co-expressed with β-catenin. The dominant-negative isoforms, _Δ30_TCF7L2-S and _Δ161_TCF7L2-S, did not activate the tested promoters, with the exception of TOP, which was activated by the thalamus-specific _Δ161_TCF7L2-S. In summary, LEF1 and TCF7L2-S acted as specific and partially β-catenin-dependent activators of the target promoters under our experimental conditions.

We then assessed the potential of LEF1, TCF7L2-S, _Δ30_TCF7L2-S, and _Δ161_TCF7L2-S to activate the cloned promoters with or without β-catenin (Fig. 5c). All of the investigated promoters responded to at least one of the LEF1/TCF7L2 factors. Among them, the *Mef2c*, *Foxp2*, *Rora*, and *Znf804a* promoters were relatively weak responders. The *Mef2* promoter was the only one that did not show any specificity for LEF1/TCF factors or isoforms and did not exhibit a requirement for β-catenin. The *Zic1* and *Etv1* promoters were activated well mainly by LEF1 (5- to 15-fold), whereas *Gbx2* and *Nr4a2* were activated well by TCF7L2-S when co-expressed with β-catenin (~5-fold and >10-fold, respectively). Finally, the *Pou4f1* promoter was activated ~10-fold by LEF1, independent of β-catenin, and >15-fold by TCF7L2-S in a β-catenin-dependent manner. In conclusion, majority of the cloned promoters were clearly responsive to LEF1, TCF7L2-S, or both. The transactivation capacity of these factors and requirement for the β-catenin cofactor differed considerably, depending on the promoter context.

## Discussion

**Fig. 6:**
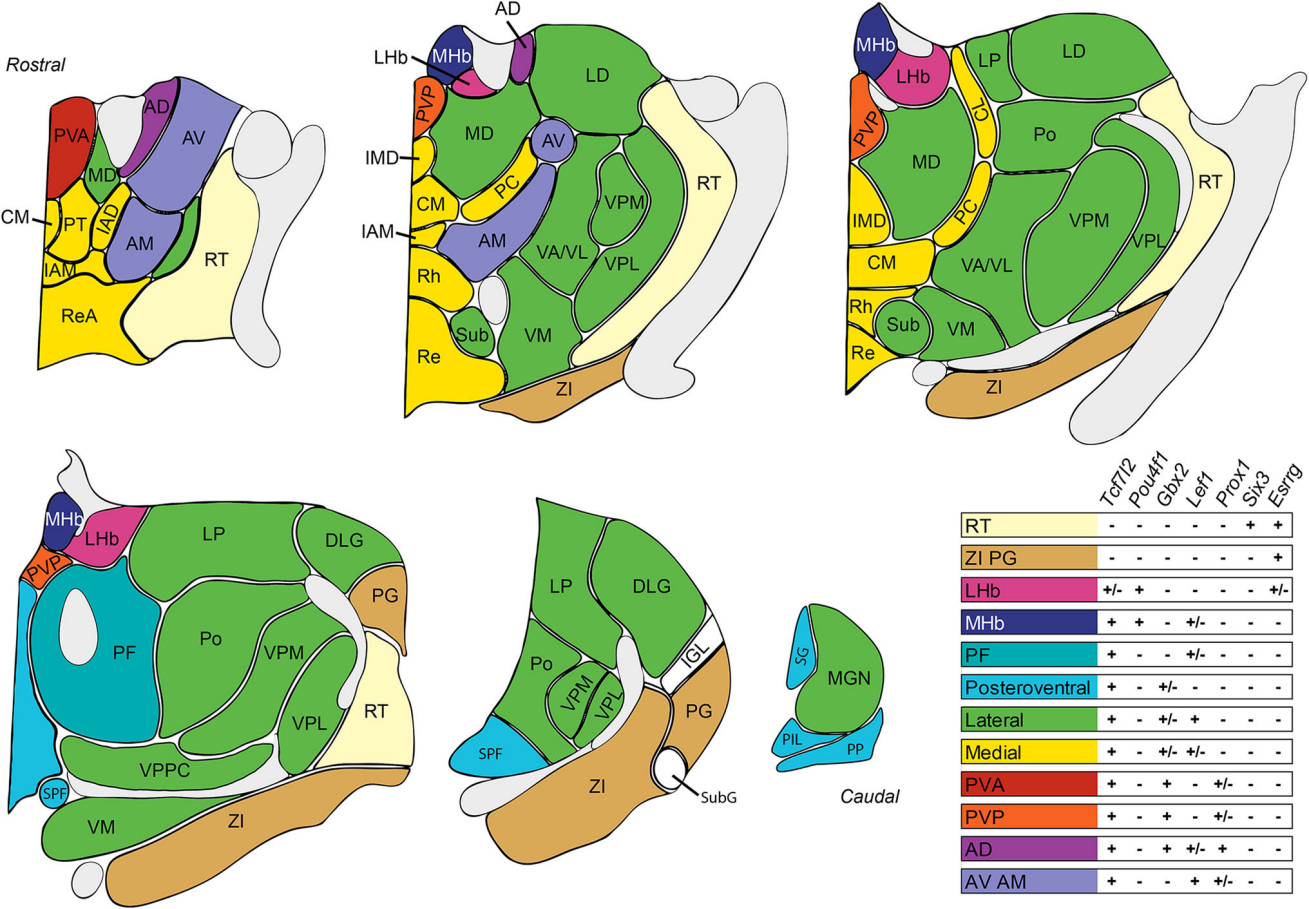
Summary of proposed organization of thalamic complex based on gene expression profiles

Based on the extensive analysis of the ISH dataset in the Allen Mouse Brain Atlas (Lein et al. 2007) with its associated visualization and data mining tools (Ng et al. 2009), we propose a new perspective on how the thalamic complex is organized with regard to shared vs. differential gene expression (Fig. 6). The present study identified transcriptional networks in the adult thalamic complex and experimentally validated in vitro the hypothesis that TCF7L2 is a master regulator of subregion-specific transcription factors in the thalamus.

### Molecular anatomy of the thalamic complex

The anatomic distinction between the thalamus and prethalamus was clearly indicated by our correlation analysis. However, we observed some deviant patterns within the thalamic structures derived from prosomere 2 (i.e., the thalamus and epithalamus or habenula), questioning some classic groupings of its nuclei. The latter were originally defined according to their topography relative to the internal medullary lamina. This landmark simply represents the way in which efferent thalamic fibers and various afferent fibers navigate across the thalamic mass and may not necessarily show a direct relationship with regard to patterning field effects.

The most striking result was obtained for the MHb, which displayed the most distinctive gene expression pattern within the thalamic complex. We observed that many transcripts are expressed exclusively in the MHb. The singular molecular profiles of the MHb and LHb are supported by a previous gene expression study (Quina et al. 2009) and reflect different functions of these two nuclei. Indeed, the MHb and LHb differentially participate in neuronal circuits (Aizawa et al. 2011). These two nuclei are currently classified as the epithalamus, a derivative of the dorsal-most progenitor domain in the alar territory of prosomere 2 that lies within the range of roof plate FGF8 signaling (Puelles et al. 2012). However, the MHb locus is the only site in prosomere 2 that contacts the insertion of the choroidal tela at the diencephalic roof, a position that is accompanied by a retarded and particularly protracted mode of neurogenesis (McAllister and Das 1977; Angevine 1970). This may explain its particular molecular characteristic. In contrast, LHb neurons have rather early birthdates and a short neurogenetic period compared with MHb and other neurons of the thalamic complex (McAllister and Das 1977; Angevine 1970). Recently, some embryologic data in the rat were reported, suggesting that the LHb (or a portion of it) may in fact originate outside the habenular territory in a caudoventral region of the thalamus, and its neurons secondarily migrate upward into the habenular domain (Puelles et al. 2015; Beretta et al. 2013). In that case, a significant part of the LHb might be regarded as not primarily epithalamic, and this observation may offer another explanation why the LHb was correlated more with prethalamic and thalamic nuclei than with MHb in our analysis. Either way, the results of our analysis support the view of intrinsic heterogeneity of the epithalamus (Geisler et al. 2003).

In our analysis, the RT, ZI, and PG were molecularly differentiated from the thalamus and from the MHb and LHb, consistent with the classic opinion that they all derive from prosomere 3 (Puelles et al. 2012). The RT, which develops at the rostral part of the prethalamus, differs from the PG and ZI with regard to the expression of a set of specific genes. The distinct molecular profile of the RT is consistent with connectivity differences between the RT and other prethalamic nuclei (Guillery et al. 1998; Mitrofanis and Mikuletic 1999; Bourassa and Deschênes 1995), possibly underpinned by their differential relative dorsoventral and anteroposterior origins within alar prosomere 3.

Partly new organizational properties of the thalamus proper emerged from our analysis. We found strong evidence of the existence of three nuclear groups, which we called medial, lateral, and posteroventral, and some small individual regions with unique gene expression patterns (Fig. 6). This especially refers to the posterior and anterior PV, PF, and AD/AM/AV, which are conventionally classified as a midline nucleus, caudal intralaminar nucleus, or anterior nuclei, respectively (Puelles et al. 2012; Van der Werf et al. 2002). Our divisions agree with groupings based on thalamic cell birthdates (i.e., neurogenetic patterns), which reportedly follow lateromedial, caudorostral, and ventrodorsal gradients (Angevine 1970; McAllister and Das 1977). The lateral, medial, and posteroventral groups appear to be homologous to rostrodorsal, caudoventral, and ventral compartments, respectively, which was previously proposed for the embryonic thalamus by González et al. (2002).

The proposed lateral group (green in Fig. 6) includes (1) nuclei that are classified as first-order (sensory) nuclei (VPL/VPM, MGN, DLG, and VA/VL), which are populated by C-type neurons and receive inputs from cortical layer 6, sensory lemniscus pathways, or deep cerebellar nuclei and project to layers 3–4 of single or adjacent cortical areas (Clascá et al. 2012); (2) associative nuclei (LP, Po, MD, MGN, VPPC, Sub, and VM) that have reciprocal connections with several cortical areas, receive afferents from the ZI, and are populated by M-type neurons that never arborize in cortical layer 4 (Deschênes et al. 1998; Power et al. 1999); (3) and the LD although this nucleus is sometimes classified with anterior nuclei because of its reciprocal connections with part of the anterior cingulate cortex (Shibata and Naito 2005). The lateral group also shares the expression of some genes with medial nuclei, LHb and PF or exclusively with the PF.

The proposed medial group (yellow in Fig. 6) includes several median and paramedian midline nuclei and rostral intralaminar nuclei, which all have reciprocal connections with the frontal cortex, send collaterals to the striatum, and receive afferents from the midbrain, brainstem, and pallidal areas (Krout et al. 2001, 2002; Groenewegen et al. 1999; Van der Werf et al. 2002).

Finally, the proposed caudally located posteroventral group (light blue in Fig. 6) includes small nuclei that are adjacent to the alar–basal boundary (SPF, PP, PIL, and SG). These nuclei receive auditory inputs and project to the amygdala and temporal part of the dorsal pallidum (Winer et al. 2002; Ledoux et al. 1987). We also found that many genes that are expressed in the posteroventral group, particularly in the PP, are shared with the IGL. This might support the thalamic origin of IGL nuclei.

As we noted above, some thalamic nuclei exhibit unique gene expression patterns, and this corresponds well to specific functions that are conferred by these nuclei. The PV serves as a nodal point between brain regions that are involved in emotional and motivational circuitry and receives strong aminergic inputs from the brainstem and peptidergic inputs from hypothalamic neurons, a property not shared by other thalamic nuclei (Li and Kirouac 2008, 2012). The PF is characterized by receiving afferents from midbrain and brainstem areas that are involved in processing sensory and motor information and has a large efferent connection with the striatum, with relatively few cortical collaterals (Van der Werf et al. 2002; Krout et al. 2002; Puelles et al. 2012). The AD is a key thalamic relay of the head-direction system, possesses a unique set of connections (via the mamillothalamic and fornix tracts), and displays characteristic electrophysiological properties (Van Groen and Wyss 1995; Taube 1995). The AM and AV also have specific functions and connectivity related to the circuit of Papez (Aggleton et al. 2010; Shibata and Naito 2005). We hypothesize that these unique thalamic nuclei either derive from distinct progenitor domains, or the mechanisms of their terminal specifications are different, possibly involving signals that spread from either the prethalamus (PV and AD/AV/AM) or pretectum (PF). These unique nuclei (and their presumptive primordia) are localized at extreme ends of the thalamus where they may be selectively accessible to morphogenetic factors. Another possibility is that these nuclei acquire their specific properties during late embryonic or early postnatal development because of singular functional conditions.

### Differential specification of thalamic complex nuclei by transcription factors

Our analysis of transcription factor expression in the thalamic complex supports the idea that its molecular identity and internal regionalization can be defined by variously overlapping combinations of active transcription factors, rather than being characterized by a single factor for each thalamic nucleus. Similar conclusions were drawn using high-throughput analysis of transcription factors in the developing subpallium, hypothalamus, and spinal cord (Del Barrio et al. 2013; Flames et al. 2007; Shimogori et al. 2010).

In the prethalamus, many transcription factors that are characteristic of early embryonic stages (e.g., *Dlx1/2/5/6*, *Gli3*, *Foxd1*, and *Lhx1* on embryonic day 11.5–13.5) are no longer expressed in the adult, suggesting that they play a role only in the early specification of prethalamic neurons (Jones and Rubenstein 2004; Nakagawa and O’Leary 2001; Puelles and Martinez 2013). However, several transcription factors, such as *Isl1*, *Six3*, *Essrg*, and *Meis2*, continue their expression in the adult mouse and human prethalamus, whereas *Pax6* is present only in mouse (Ehrman et al. 2013; Lavado et al. 2008; Toresson et al. 2000; Pratt et al. 2000). In the epithalamus, *Pou4f1* is specifically and highly expressed, and its involvement in the activation of habenula-specific genes has been previously reported (Quina et al. 2009). Expression of this gene is also conserved in the human epithalamus. Thalamus-specific transcription factors show diverse and overlapping expression patterns that align with the proposed nuclear groups. For example, *Foxp1*, *Foxp2*, *Gbx2*, and *Tox* are all expressed in the medial group. Whereas *Foxp2* and *Gbx2* are also expressed in some of the lateral nuclei, *Tox* and *Foxp1* are not expressed in any of them. All anterior nuclei are characterized by the expression of *Prox1*, but each of these nuclei also expresses some specific transcription factors that are not expressed by others. For example, the AD selectively expresses *Id4* and *Arnt2*, whereas AM expresses *Rreb1* and *Foxp1*. Neurons in the PF strongly express *Etv1* but do not express *Zic1* or *Id4*, which are expressed in many thalamic nuclei. The PV is characterized by the high expression of *Hopx*, *Tox*, and *Gbx2*. Several transcription factor genes are also broadly expressed in the adult mouse and human thalamus, particularly *Tcf7l2* and *Lef1*, the expression of which in the mouse thalamic area is maintained throughout development and in adulthood (Nagalski et al. 2013). *Tcf7l2* is expressed in all thalamic and epithalamic nuclei, and *Lef1* is expressed in most nuclei, with the exception of the PV and posteroventral group. We suppose that the transcription factors that are specific for different groups of nuclei or unique nuclei can serve as molecular markers that stably and selectively demarcate these areas and might participate in the regionalization of the thalamic complex and its parts.

### TCF7L2 as a terminal selector in prosomere 2

The aforementioned TCF7L2 and LEF1 are effectors of Wnt/β-catenin signaling. Our results showed that the promoters of some thalamus- and epithalamus-specific transcription factors, namely *Etv1*, *Foxp2*, *Gbx2*, *Mef2c*, *Nr4a2*, *Pou4f1*, *Rora*, *Zic1*, and *Znf804a*, are responsive to the thalamic TCF7L2-S isoform and/or LEF1, at least using a cell line. Several of these transcription factors are known to be involved in brain development. POU4F1 (alias BRN3A) together with NR4A2 (alias NURR1) regulates the coordinated expression of habenula-enriched genes, and habenular connections are lost in *Pou4f1*^−*/*−^ embryos (Quina et al. 2009). GBX2 is a marker of postmitotic thalamic neurons, and the thalamus is disrupted in *Gbx2*^−*/*−^ embryos (Li et al. 2012; Chen et al. 2009). PROX1 has been shown to regulate adult neurogenesis in the hippocampus as a target of the Wnt/β-catenin pathway (Karalay et al. 2011). We recently identified other genes (i.e., *Cacna1g*, *Kcna6*, *Calb2*, *Gabra3*, *Cacna2d2*, and *Kcnh8*) with conserved LEF/TCF motifs in their promoter regions that were highly expressed in the thalamus and regulated by β-catenin (Wisniewska et al. 2010, 2012). They are likely directly regulated by TCF7L2 as a β-catenin effector. Data in the Allen Mouse Brain Atlas indicate that the expression of these genes is restricted to different sets of thalamic nuclei. Given the structural and functional complexity of the thalamus, it is rather implausible that TCF7L2 can regulate the same genetic programs by itself in all of the nuclei. Gene regulation by TCF7L2 is known to be context-dependent (Frietze et al. 2012; Boj et al. 2012). We hypothesize that the thalamus-specific isoform TCF7L2-S and/or LEF1 can eventually regulate various thalamic and epithalamic transcription factors, with expression patterns restricted to some areas. TCF7L2 and LEF1 may represent terminal selector genes of thalamic neurons, analogous to AST-1 in dopaminergic neurons (Flames and Hobert 2009) and UNC-3 in cholinergic motor neurons (Kratsios et al. 2012) of *Cenorhabditis elegans* or Pet-1 in mouse serotonergic neurons (Liu et al. 2010; Alonso et al. 2013). However, our hypothesis has yet to be confirmed with further studies in knockout mouse models.

### Possible relevance to schizophrenia and other neuropsychiatric disorders

Several lines of evidence indicate thalamic dysfunction and thalamocortical disconnectivity in psychiatric conditions such as schizophrenia (Behrendt 2003; Woodward et al. 2012; Alelú-Paz and Giménez-Amaya 2008; Ellison-Wright and Bullmore 2010; Pratt and Morris 2015; Popken et al. 2000; Byne et al. 2009; Kumari et al. 2010), bipolar disorder (Radenbach et al. 2010), major depression (Young et al. 2004; Greicius et al. 2007; Li et al. 2013), and autism spectrum disorder (Nair et al. 2013). The aforementioned disorders are considered to be of neurodevelopmental origin; therefore, understanding the development of the thalamic complex may help to reveal their etiology. Two of the transcription factors that were identified here to be specific for thalamic subregions have been associated with schizophrenia, bipolar disorder and autism in genome-wide association studies: TCF7L2 (Alkelai et al. 2012; Iossifov et al. 2014; Winham et al. 2014) and ZNF804A (Consortium SWGotPG 2014; Williams et al. 2011a, b; O’Donovan et al. 2008). In particular, the rs1344706 single nucleotide polymorphism (SNP) in intron two of *ZNF804A* was the first variant to reach an unequivocal genome-wide significance for schizophrenia (O’Donovan et al. 2008) with later meta-analyses confirming the association and extending it to a broader psychosis phenotype (Zhu et al. 2014; Sun et al. 2015; Williams et al. 2011a, b). Interestingly, the subregions in the mouse thalamic complex where *Znf804a* is expressed overlap with the regions that are affected in schizophrenic patients, i.e.: the prethalamic reticular nucleus (Pratt and Morris 2015), MD (Alelú-Paz and Giménez-Amaya 2008; Byne et al. 2009), pulvinar nucleus (Byne et al. 2009; Kumari et al. 2010) considered to be an equivalent of the LP in rodents (Baldwin et al. 2011), and MGN (Kumari et al. 2010). The function of this transcription factor and the potential mechanism by which it might increase risk for schizophrenia is still not known. Morphometric analysis of schizophrenic brains revealed a possible effect of *ZNF804A* genetic variation on the frontal cortical regions and thalamus (Nenadic et al. 2015), suggesting an involvement of ZNF804A in the development of these areas of the brain. Even less is known about the relationship between TCF7L2 and psychopathologies, except that *Tcf7l2* haploinsufficient mice show anxiety-like behavior and altered fear learning (Savic et al. 2011). It is important to note here that TCF7L2 (Transcription Factor 7-Like 2, HMG-box) has been initially abbreviated as TCF4 (T cell factor 4), and it should not be confused with a basic helix-loop-helix transcription factor TCF4 (Transcription Factor 4, alias E2-2, ITF2), which is also associated with schizophrenia. Further research is needed to elucidate the role of ZNF804A and TCF7L2 in thalamus development and in the etiology of psychiatric disorders.

## Conclusion

We provided an in vivo contextual framework that will aid future studies by consolidating the diversity of thalamic nuclei into manageable cardinal classes with specific gene expression profiles. These may prove useful for designing tools to explore thalamic regionalization, connections, physiological properties, and gene functions. Such studies may ultimately allow the selective manipulation of individual nuclei and a more detailed analysis of their contribution to mammalian physiology and behavior.

## Experimental procedures

### Mapping of genetic relationship between thalamic complex nuclei and selection of representative transcription factors

The mapping was based on a systematic analysis of gene expression in the thalamic complex using Allen Brain Atlas databases. To identify distinct subdivisions of the thalamic complex based on spatial gene expression patterns, we used the AGEA, which is an open-access, three-dimensional atlas of the adult C57BL/6J mouse strain that provides information about the degree of gene expression similarity between regions of the mouse brain. To examine correlations in gene expression between thalamic nuclear units and groups, we selected points (seed voxels) in the center of each of 24 thalamic, epithalamic, and prethalamic nuclei by means of positioning a cross-hair cursor on the Nissl reference images that are displayed in the AGEA correlation maps. Pearson correlation coefficients were not randomly collected from five different points that were localized in different anteroposterior and dorsoventral regions within the anatomically defined area of each of the selected nuclei. For the nuclei with known subregions, like MGN and MD, we endeavor to collect correlation from all this subregions. All of the correlation data are available in Supplementary Material 1. Mean correlations were then clustered using the Average linkage and Manhattan distance functions according to Hawrylycz et al. (2010). The correlations were converted to Z-scores and then to *P* values as described (Motulsky 2013). Using Gene Finder tools in the AGEA application, we filtered candidate genes that showed regional specificity. To select transcription factors that are expressed in the adult thalamic region, we used the “Anatomic search” and “Neuroblast” tools in near-adult brains (postnatal day 28) of the Developing Mouse Brain Atlas (http://developingmouse.brain-map.org; accessed July 30, 2014). This transcription factor gene list was further verified with coronal sections of the adult mouse brain available in the Allen Mouse Brain Atlas (Lein et al. 2007), and the level of ISH signal was visually estimated as high (3), medium (2), or low level/scattered (1) expression or undetected (0). To select transcription factors enriched in the adult human prethalamus, epithalamus and thalamus we used “Anatomic search” tool from Human Brain Atlas (Hawrylycz et al. 2012). From gene list we selected transcription factors using DAVID functional annotation (Huang et al. 2009) and further verified the expression pattern of selected genes based on microarray data available in the Human Brain Atlas.

### Bioinformatics

The analysis of regulatory element/transcription factor binding sites was performed as described previously (Wisniewska et al. 2012). For details of the screening procedure, see the online Supplementary Material.

### Luciferase assay

Luciferase assays were performed as described previously (Wisniewska et al. 2010) using HeLa cells. See Supplementary Material for further details, including sequence analysis and promoter cloning.

## Electronic supplementary material

Below is the link to the electronic supplementary material.

Supplementary material 1 (PDF 533 kb)

Supplementary material 2 (XLS 258 kb)

Supplementary material 3 (XLS 284 kb)

Supplementary material 4 (XLS 86 kb)

Supplementary material 5 (PDF 460 kb)

## Abbreviations

AD: Anterodorsal thalamic nucleus
AM: Anteromedial thalamic nucleus
AV: Anteroventral thalamic nucleus
CL: Centrolateral thalamic nucleus
CM: Central medial thalamic nucleus
DLG: Dorsal lateral geniculate nucleus
IAD: Interanterodorsal thalamic nucleus
IMD: Intermediodorsal thalamic nucleus
LD: Laterodorsal thalamic nucleus
LHb: Lateral habenula
LP: Lateral posterior thalamic nucleus
MD: Mediodorsal thalamic nucleus
MGD: Medial geniculate nucleus, dorsal part
MGM: Medial geniculate nucleus, medial part
MGN: Medial geniculate nucleus
MGV: Medial geniculate nucleus, ventral part
MHb: Medial habenula
PF: Parafascicular thalamic nucleus
PG: Pregeniculate nucleus
PIL: Posterior intralaminar thalamic nucleus
Po: Posterior thalamic nuclear group
PP: Peripeduncular nucleus
PT: Paratenial thalamic nucleus
PV: Paraventricular thalamic nucleus
PVA: Paraventricular thalamic nucleus, anterior part
PVP: Paraventricular thalamic nucleus, posterior part
Re: Reuniens thalamic nucleus
ReA: Reuniens thalamic nucleus, anterior part
Rh: Rhomboid thalamic nucleus
RT: Reticular thalamic nucleus
SG: Suprageniculate thalamic nucleus
SPF: Subparafascicular thalamic nucleus
Sub: Submedius thalamic nucleus
VA/VL: Ventral anterior/ventral lateral thalamic nuclei
VM: Ventromedial thalamic nucleus
VPL/VPM: Ventral posterolateral/ventral posteromedial thalamic nucleus
VPPC: Ventral posterior nucleus of the thalamus, parvicellular part
ZI: Zona incerta
