# Supplementary material for: Molecular anatomy of the thalamic complex and the underlying transcription factors
Source: Brain Struct Funct. 2015 May 12;221:2493–510. doi: 10.1007/s00429-015-1052-5 (PMC4884203; doi:10.1007/s00429-015-1052-5)
Supplement: Supplementary file 1 — Supplementary material 1 (PDF 533 kb) [file 429_2015_1052_MOESM1_ESM.pdf]

Supplementary Data 1 - AGEA analysis

Accessed 5 march 2013

AGEA hyperlink

Position of seed voxel	Seed voxel	Pearson correlation values					Mean correlation	Standard Deviation
map.org/agea?seed=P	**DLG**	1.0000	0.9863	0.9262	1.0000	0.9507	0.9726	0.0329
	PG	0.8244	0.7772	0.8096	0.7616	0.7660	0.7878	0.0278
	MGN	0.7590	0.8041	0.7299	0.8624	0.8569	0.8025	0.0586
	VPL/VPM	0.9325	0.9236	0.9235	0.9343	0.9336	0.9295	0.0055
	VL/VA	0.9186	0.9234	0.9053	0.9090	0.9137	0.9140	0.0073
	VM	0.8777	0.8820	0.8898	0.8789	0.8994	0.8856	0.0091
	MD	0.9016	0.8819	0.9050	0.9099	0.9079	0.9013	0.0113
	LD	0.8191	0.8319	0.8191	0.9059	0.9130	0.8578	0.0475
	Po	0.9080	0.8746	0.8573	0.8902	0.9012	0.8863	0.0205
	AD	0.8115	0.8482	0.8482	0.8128	0.8467	0.8335	0.0195
	AM	0.8561	0.8313	0.8319	0.8248	0.8817	0.8452	0.0237
	AV	0.8552	0.8446	0.8918	0.8849	0.8379	0.8629	0.0242
	LP	0.8390	0.8757	0.9039	0.8790	0.8492	0.8694	0.0258
	PV	0.7520	0.8061	0.6279	0.7802	0.7863	0.7505	0.0712
	PT	0.7441	0.7445	0.7832	0.8151	0.7441	0.7662	0.0321
	IMD	0.8538	0.8681	0.8664	0.7688	0.7748	0.8264	0.0502
	Re	0.8078	0.8078	0.8078	0.8125	0.8138	0.8099	0.0030
	CM	0.8202	0.8415	0.8106	0.8167	0.8411	0.8260	0.0144
	PF	0.8068	0.7858	0.7776	0.7858	0.7861	0.7884	0.0109
	RT	0.8640	0.8698	0.7610	0.8207	0.8565	0.8344	0.0453
	MHb	0.6304	0.5775	0.6292	0.5333	0.5610	0.5863	0.0428
	LHb	0.6292	0.6460	0.7185	0.7287	0.7628	0.6970	0.0570
	ZI	0.7475	0.7820	0.7675	0.7706	0.8556	0.7846	0.0416
	SPF	0.8029	0.7724	0.7896	0.8674	0.8270	0.8119	0.0369

Position of seed voxel	Seed voxel	Pearson correlation values					Mean correlation	Standard Deviation
	DLG	0.7792	0.7846	0.8091	0.8429	0.8148	0.8061	0.0256
map.org/agea?seed=P	**PG**	1.0000	0.9812	0.9808	0.9496	0.9808	0.9785	0.0181
	MGN	0.7947	0.7340	0.8230	0.7248	0.7248	0.7603	0.0456
	VPL/VPM	0.7743	0.7663	0.8149	0.8270	0.7625	0.7890	0.0298
	VL/VA	0.7841	0.7643	0.7761	0.7596	0.7781	0.7724	0.0102
	VM	0.8204	0.8091	0.7695	0.7815	0.7953	0.7952	0.0205
	MD	0.7929	0.7957	0.7734	0.7731	0.7757	0.7822	0.0112
	LD	0.8120	0.8010	0.8134	0.7949	0.7888	0.8020	0.0107
	Po	0.7878	0.7563	0.7590	0.8194	0.8378	0.7921	0.0362
	AD	0.8146	0.8206	0.8146	0.8189	0.8206	0.8179	0.0031
	AM	0.8627	0.7493	0.7858	0.7493	0.7916	0.7877	0.0463
	AV	0.7869	0.8206	0.7733	0.7515	0.7733	0.7811	0.0255
	LP	0.8068	0.7768	0.7702	0.7708	0.8033	0.7856	0.0180
	PV	0.8056	0.8103	0.7999	0.8242	0.8123	0.8105	0.0090
	PT	0.8171	0.8284	0.8286	0.8149	0.8171	0.8212	0.0067
	IMD	0.8170	0.8139	0.7887	0.7844	0.7837	0.7975	0.0165
	Re	0.8152	0.8165	0.8098	0.8098	0.8452	0.8193	0.0148
	CM	0.7911	0.7849	0.8132	0.8264	0.7865	0.8004	0.0184
	PF	0.8583	0.8583	0.8156	0.8345	0.8424	0.8418	0.0179
	RT	0.8685	0.8814	0.8727	0.8689	0.8605	0.8704	0.0076
	MHb	0.5602	0.6166	0.6431	0.6658	0.6276	0.6227	0.0395
	LHb	0.9000	0.8912	0.8792	0.8633	0.8370	0.8741	0.0249
	ZI	0.9376	0.9310	0.9236	0.9212	0.9200	0.9267	0.0075
	SPF	0.9010	0.8971	0.8809	0.9236	0.9085	0.9022	0.0156

Position of seed voxel	Seed voxel	Pearson correlation values					Mean correlation	Standard Deviation
	DLG	0.9125	0.8919	0.9157	0.9079	0.8684	0.8993	0.0195
	PG	0.8011	0.8319	0.8011	0.8319	0.8011	0.8134	0.0169
map.org/agea?seed=P	**MGN**	0.9489	0.9706	0.9603	0.9593	0.9464	0.9571	0.0097
	VPL/VPM	0.8898	0.8838	0.8894	0.8931	0.8841	0.8880	0.0040
	VL/VA	0.8836	0.8936	0.8817	0.8867	0.8836	0.8858	0.0047
	VM	0.8720	0.8810	0.8760	0.8720	0.8938	0.8790	0.0091
	MD	0.9090	0.9149	0.8999	0.9012	0.9102	0.9070	0.0063

	LD	0.8153	0.8737	0.8861	0.8804	0.8704	0.8652	0.0285
	Po	0.8979	0.9173	0.9292	0.9426	0.9358	0.9246	0.0176
	AD	0.8241	0.7855	0.7696	0.8118	0.7184	0.7819	0.0414
	AM	0.8033	0.8570	0.8482	0.8791	0.8871	0.8549	0.0329
	AV	0.8681	0.8168	0.7095	0.7710	0.7680	0.7867	0.0593
	LP	0.9293	0.9307	0.9359	0.9241	0.9331	0.9306	0.0044
	PV	0.7199	0.7416	0.7856	0.8152	0.7862	0.7697	0.0383
	PT	0.7341	0.7335	0.8033	0.7817	0.7898	0.7685	0.0326
	IMD	0.8099	0.8238	0.8406	0.8587	0.8882	0.8442	0.0306
	Re	0.8003	0.8275	0.8423	0.8089	0.7866	0.8131	0.0220
	CM	0.8425	0.8555	0.8817	0.8825	0.8820	0.8688	0.0187
	PF	0.8795	0.8598	0.8459	0.7992	0.7896	0.8348	0.0389
	RT	0.7433	0.7535	0.7522	0.8126	0.8434	0.7810	0.0444
	MHb	0.5950	0.5738	0.6049	0.6136	0.5853	0.5945	0.0157
	LHb	0.6700	0.7001	0.6408	0.6414	0.6250	0.6555	0.0298
	ZI	0.8583	0.8125	0.7817	0.8772	0.8485	0.8356	0.0382
	SPF	0.8907	0.8570	0.9069	0.7764	0.8022	0.8466	0.0561

Position of seed voxel	Seed voxel	Pearson correlation values					Mean correlation	Standard Deviation
	DLG	0.9419	0.9238	0.9034	0.9339	0.9416	0.9289	0.0161
	PG	0.7898	0.6614	0.7973	0.7642	0.7310	0.7487	0.0553
	MGN	0.8007	0.8178	0.8652	0.8932	0.8726	0.8499	0.0390
map.org/agea?seed=P	**VPL/VPM**	0.9894	0.9880	0.9944	0.9839	0.9776	0.9867	0.0063
	VL/VA	0.9473	0.8925	0.9182	0.8805	0.9168	0.9111	0.0259
	VM	0.9038	0.8684	0.8680	0.8883	0.8773	0.8812	0.0151
	MD	0.9144	0.9144	0.9279	0.9100	0.9193	0.9172	0.0068
	LD	0.9410	0.9523	0.9556	0.9241	0.9426	0.9431	0.0123
	Po	0.8811	0.8899	0.9039	0.8899	0.8604	0.8850	0.0160
	AD	0.8404	0.8226	0.8548	0.8147	0.8871	0.8439	0.0287
	AM	0.7879	0.7988	0.8142	0.7911	0.8221	0.8028	0.0148
	AV	0.8267	0.8199	0.8639	0.8721	0.8068	0.8379	0.0286
	LP	0.8308	0.8588	0.8722	0.8401	0.8588	0.8521	0.0165
	PV	0.6566	0.6869	0.7274	0.8012	0.7166	0.7177	0.0542
	PT	0.8041	0.7971	0.7342	0.7357	0.7342	0.7611	0.0362
	IMD	0.8351	0.8519	0.8683	0.8659	0.8091	0.8461	0.0245
	Re	0.7758	0.7358	0.7159	0.7860	0.7758	0.7579	0.0303
	CM	0.7989	0.7989	0.8134	0.7999	0.8077	0.8038	0.0065
	PF	0.7943	0.8147	0.7730	0.7628	0.7766	0.7843	0.0205
	RT	0.7810	0.7720	0.8466	0.7999	0.7885	0.7976	0.0292
	MHb	0.4711	0.5259	0.5489	0.5723	0.5843	0.5405	0.0448
	LHb	0.7125	0.6900	0.7377	0.7395	0.7334	0.7226	0.0212
	ZI	0.7220	0.7302	0.7689	0.7592	0.7522	0.7465	0.0198
	SPF	0.8442	0.8362	0.8044	0.8021	0.7842	0.8142	0.0251

Position of seed voxel	Seed voxel	Pearson correlation values					Mean correlation	Standard Deviation
	DLG	0.9294	0.9293	0.9087	0.9061	0.9216	0.9190	0.0111
	PG	0.8528	0.7805	0.8296	0.8272	0.7984	0.8177	0.0284
	MGN	0.8006	0.7742	0.8006	0.8200	0.8029	0.7997	0.0164
	VPL/VPM	0.9378	0.9432	0.9442	0.9369	0.9440	0.9412	0.0036
map.org/agea?seed=P	**VL/VA**	0.9881	0.9915	1.0000	0.9703	0.9832	0.9866	0.0110
	VM	0.9327	0.9331	0.9297	0.9205	0.8781	0.9188	0.0233
	MD	0.9287	0.9363	0.9178	0.9206	0.9369	0.9281	0.0088
	LD	0.8559	0.9402	0.8926	0.9004	0.9183	0.9015	0.0314
	Po	0.8899	0.8876	0.8785	0.8899	0.9072	0.8906	0.0104
	AD	0.8740	0.8798	0.7449	0.8740	0.8481	0.8442	0.0568
	AM	0.8985	0.8893	0.8465	0.8448	0.8626	0.8683	0.0246
	AV	0.8355	0.8643	0.8514	0.9118	0.8540	0.8634	0.0290
	LP	0.8788	0.8788	0.8655	0.8797	0.8974	0.8800	0.0114
	PV	0.7862	0.8121	0.8517	0.7893	0.7704	0.8019	0.0315
	PT	0.7806	0.7482	0.8428	0.8485	0.7806	0.8001	0.0436
	IMD	0.8299	0.8723	0.8883	0.8836	0.8861	0.8720	0.0243
	Re	0.7602	0.7936	0.8179	0.8367	0.7764	0.7970	0.0308
	CM	0.8591	0.8735	0.8669	0.8687	0.8839	0.8704	0.0091
	PF	0.8099	0.7968	0.7766	0.8182	0.7989	0.8001	0.0157

	RT	0.8091	0.8004	0.8357	0.8671	0.8623	0.8349	0.0302
	MHb	0.5039	0.5592	0.5835	0.5962	0.6536	0.5793	0.0546
	LHb	0.8242	0.7541	0.7575	0.7355	0.7987	0.7740	0.0363
	ZI	0.7986	0.7876	0.7805	0.8601	0.8253	0.8104	0.0326
	SPF	0.8236	0.8006	0.8390	0.8600	0.7665	0.8179	0.0360

**Position of seed voxel**	**Seed voxel**	**Pearson correlation values**					**Mean correlation**	**Standard Deviation**
	DLG	0.8925	0.8869	0.8857	0.8822	0.8709	0.8836	0.0080
	PG	0.8333	0.7953	0.7687	0.8184	0.8004	0.8032	0.0245
	MGN	0.8538	0.8178	0.8381	0.7012	0.8178	0.8057	0.0604
	VPL/VPM	0.8832	0.8826	0.8958	0.9104	0.8941	0.8932	0.0114
	VL/VA	0.9328	0.9228	0.9319	0.9092	0.9185	0.9230	0.0098
map.org/agea?seed=P	**VM**	0.9569	0.9627	0.9545	0.9262	0.9349	0.9470	0.0157
	MD	0.9294	0.9011	0.9014	0.9294	0.9097	0.9142	0.0143
	LD	0.8894	0.8934	0.8969	0.8863	0.9101	0.8952	0.0092
	Po	0.9044	0.8101	0.9108	0.9066	0.9037	0.8871	0.0431
	AD	0.8498	0.8254	0.8498	0.8285	0.7470	0.8201	0.0424
	AM	0.8950	0.8688	0.8631	0.8828	0.8621	0.8744	0.0142
	AV	0.8487	0.8506	0.8557	0.8506	0.8487	0.8509	0.0029
	LP	0.8868	0.8913	0.8918	0.8911	0.9129	0.8948	0.0103
	PV	0.8040	0.7776	0.7805	0.8270	0.8139	0.8006	0.0213
	PT	0.8671	0.8654	0.8671	0.8408	0.8073	0.8495	0.0261
	IMD	0.8847	0.8841	0.9110	0.9216	0.9252	0.9053	0.0198
	Re	0.8497	0.8662	0.8669	0.8856	0.8794	0.8696	0.0138
	CM	0.8836	0.8855	0.9096	0.9070	0.8703	0.8912	0.0167
	PF	0.8250	0.8181	0.8195	0.8122	0.8316	0.8213	0.0073
	RT	0.7879	0.7929	0.8576	0.8797	0.7581	0.8152	0.0511
	MHb	0.6622	0.6819	0.6951	0.6104	0.5684	0.6436	0.0530
	LHb	0.7511	0.7699	0.7910	0.7828	0.7900	0.7770	0.0167
	ZI	0.8198	0.7755	0.8088	0.8042	0.7950	0.8007	0.0167
	SPF	0.8700	0.9029	0.9063	0.9077	0.8999	0.8974	0.0156

**Position of seed voxel**	**Seed voxel**	**Pearson correlation values**					**Mean correlation**	**Standard Deviation**
	DLG	0.8904	0.9145	0.9174	0.9058	0.9208	0.9098	0.0122
	PG	0.7941	0.7941	0.7556	0.8101	0.7442	0.7796	0.0282
	MGN	0.8648	0.9008	0.9125	0.9081	0.9273	0.9027	0.0233
	VPL/VPM	0.9087	0.9154	0.9068	0.9104	0.9089	0.9100	0.0033
	VL/VA	0.9512	0.9437	0.9512	0.9335	0.9301	0.9419	0.0098
	VM	0.9313	0.9438	0.9369	0.9326	0.9369	0.9363	0.0049
map.org/agea?seed=P	**MD**	0.9562	0.9905	0.9847	0.9767	0.9744	0.9765	0.0130
	LD	0.9478	0.9466	0.9469	0.9272	0.9080	0.9353	0.0175
	Po	0.9443	0.9405	0.9448	0.9326	0.9286	0.9382	0.0072
	AD	0.8660	0.8789	0.9789	0.7604	0.8325	0.8633	0.0793
	AM	0.9120	0.9003	0.9210	0.9164	0.9081	0.9116	0.0079
	AV	0.9114	0.8744	0.8857	0.8786	0.8857	0.8872	0.0144
	LP	0.8886	0.9140	0.9216	0.9362	0.9279	0.9177	0.0182
	PV	0.7736	0.7885	0.8233	0.8545	0.8517	0.8183	0.0365
	PT	0.8191	0.8908	0.8630	0.8908	0.8908	0.8709	0.0314
	IMD	0.9369	0.9389	0.9310	0.8377	0.8982	0.9085	0.0429
	Re	0.8899	0.8661	0.8423	0.9029	0.8493	0.8701	0.0259
	CM	0.8966	0.9070	0.9473	0.9380	0.9473	0.9272	0.0238
	PF	0.8803	0.8662	0.8585	0.8357	0.8097	0.8501	0.0278
	RT	0.8891	0.9077	0.7966	0.8110	0.7869	0.8383	0.0560
	MHb	0.5027	0.5693	0.5889	0.5962	0.6693	0.5853	0.0597
	LHb	0.8465	0.8678	0.7759	0.6894	0.7881	0.7935	0.0698
	ZI	0.7871	0.7905	0.7803	0.8184	0.7808	0.7914	0.0157
	SPF	0.8079	0.8643	0.8869	0.8116	0.8562	0.8454	0.0344

**Position of seed voxel**	**Seed voxel**	**Pearson correlation values**					**Mean correlation**	**Standard Deviation**
	DLG	0.9117	0.9170	0.9036	0.9083	0.8824	0.9046	0.0133
	PG	0.8199	0.8199	0.7967	0.7493	0.8333	0.8038	0.0332
	MGN	0.8394	0.7903	0.8633	0.8464	0.7952	0.8269	0.0324
	VPL/VPM	0.8917	0.8956	0.9091	0.9246	0.9317	0.9105	0.0175
	VL/VA	0.9495	0.9539	0.9353	0.9371	0.9174	0.9386	0.0143

Position of seed voxel	Seed voxel	Pearson correlation values					Mean correlation	Standard Deviation
	VM	0.8729	0.9093	0.9145	0.8825	0.8656	0.8890	0.0219
	MD	0.8932	0.9181	0.9216	0.8960	0.9074	0.9073	0.0127
map.org/agea?seed=P	**LD**	0.9365	0.9543	0.9090	0.9282	0.9880	0.9432	0.0299
	Po	0.8840	0.8832	0.9021	0.9098	0.8758	0.8910	0.0143
	AD	0.8626	0.8267	0.8519	0.8626	0.8864	0.8580	0.0216
	AM	0.8427	0.8969	0.8755	0.9162	0.9009	0.8864	0.0284
	AV	0.9295	0.8518	0.8578	0.9302	0.9586	0.9056	0.0479
	LP	0.8814	0.9037	0.8705	0.8774	0.9094	0.8885	0.0171
	PV	0.7583	0.7617	0.8251	0.8204	0.8144	0.7960	0.0331
	PT	0.7635	0.7647	0.8191	0.8324	0.8097	0.7979	0.0319
	IMD	0.8642	0.8726	0.8807	0.8589	0.7523	0.8457	0.0529
	Re	0.7484	0.8081	0.8684	0.8890	0.8283	0.8284	0.0550
	CM	0.8415	0.8642	0.8783	0.8896	0.8832	0.8714	0.0191
	PF	0.8698	0.8941	0.8634	0.8357	0.8389	0.8604	0.0240
	RT	0.7445	0.7533	0.8673	0.9057	0.7625	0.8067	0.0744
	MHb	0.6602	0.6743	0.6814	0.6471	0.6453	0.6617	0.0161
	LHb	0.6774	0.6823	0.7659	0.7559	0.8512	0.7465	0.0713
	ZI	0.7919	0.8101	0.8559	0.8634	0.8360	0.8315	0.0302
	SPF	0.8183	0.8542	0.8765	0.8261	0.8482	0.8447	0.0232

Position of seed voxel	Seed voxel	Pearson correlation values					Mean correlation	Standard Deviation
	DLG	0.8961	0.8922	0.9063	0.9103	0.9080	0.9026	0.0079
	PG	0.7746	0.8198	0.8308	0.8624	0.8709	0.8317	0.0383
	MGN	0.9004	0.9035	0.9105	0.9087	0.9105	0.9067	0.0045
	VPL/VPM	0.8934	0.9302	0.9075	0.8793	0.8815	0.8984	0.0210
	VL/VA	0.8784	0.8899	0.9004	0.9013	0.8786	0.8897	0.0112
	VM	0.8888	0.9046	0.8801	0.8895	0.9046	0.8935	0.0108
	MD	0.9254	0.9286	0.9260	0.9317	0.9337	0.9291	0.0036
	LD	0.8936	0.8973	0.8845	0.8955	0.8409	0.8824	0.0237
map.org/agea?seed=P	**Po**	0.9916	0.9862	0.9508	0.9476	0.9799	0.9712	0.0206
	AD	0.8204	0.8570	0.8204	0.8570	0.8357	0.8381	0.0183
	AM	0.8798	0.8979	0.8949	0.8879	0.8932	0.8907	0.0071
	AV	0.8740	0.9081	0.8858	0.9054	0.8948	0.8936	0.0141
	LP	0.9375	0.9389	0.9639	0.9546	0.9749	0.9540	0.0161
	PV	0.7920	0.8304	0.8502	0.8942	0.8989	0.8531	0.0448
	PT	0.8163	0.8020	0.8132	0.8708	0.8479	0.8300	0.0285
	IMD	0.8726	0.9181	0.9125	0.9136	0.8952	0.9024	0.0188
	Re	0.8351	0.8560	0.8635	0.8560	0.8764	0.8574	0.0150
	CM	0.9134	0.9397	0.9210	0.9137	0.9210	0.9218	0.0107
	PF	0.8598	0.8472	0.8379	0.8969	0.8859	0.8655	0.0252
	RT	0.8613	0.8016	0.8233	0.8390	0.8002	0.8251	0.0259
	MHb	0.6392	0.6753	0.5916	0.6075	0.6177	0.6263	0.0324
	LHb	0.7955	0.6889	0.7755	0.7543	0.6987	0.7426	0.0470
	ZI	0.8093	0.8648	0.8146	0.8263	0.7805	0.8191	0.0306
	SPF	0.8877	0.9041	0.9183	0.8830	0.8997	0.8986	0.0140

Position of seed voxel	Seed voxel	Pearson correlation values					Mean correlation	Standard Deviation
	DLG	0.8166	0.8297	0.8156	0.8128	0.8135	0.8176	0.0069
	PG	0.7809	0.7611	0.8252	0.7776	0.7780	0.7846	0.0240
	MGN	0.7345	0.7345	0.7231	0.7366	0.7403	0.7338	0.0064
	VPL/VPM	0.8316	0.8262	0.8295	0.8386	0.8253	0.8302	0.0053
	VL/VA	0.8481	0.8497	0.8326	0.8447	0.8549	0.8460	0.0083
	VM	0.8154	0.8156	0.7879	0.8160	0.8254	0.8121	0.0141
	MD	0.8193	0.8284	0.8187	0.8165	0.8284	0.8223	0.0057
	LD	0.8708	0.8798	0.8576	0.8559	0.8678	0.8664	0.0099
	Po	0.8106	0.7777	0.7782	0.7784	0.7831	0.7856	0.0141
map.org/agea?seed=P	**AD**	0.9331	0.9679	1.0000	0.9679	0.9281	0.9594	0.0294
	AM	0.8583	0.8468	0.8845	0.8940	0.8772	0.8722	0.0193
	AV	0.9457	0.8637	0.8489	0.9051	0.9184	0.8964	0.0397
	LP	0.8298	0.8319	0.8041	0.8029	0.8264	0.8190	0.0143
	PV	0.7533	0.7941	0.8858	0.8448	0.7985	0.8153	0.0510
	PT	0.8035	0.8012	0.8191	0.8012	0.8035	0.8057	0.0076
	IMD	0.8115	0.8086	0.8092	0.8147	0.8166	0.8121	0.0035
	Re	0.8380	0.8211	0.8361	0.8425	0.8019	0.8279	0.0166
	CM	0.8436	0.8439	0.8298	0.8188	0.8221	0.8316	0.0118

	PF	0.8237	0.8224	0.8284	0.8312	0.8295	0.8270	0.0038
	RT	0.8166	0.8453	0.8407	0.8456	0.8390	0.8374	0.0120
	MHb	0.5842	0.8254	0.6525	0.6614	0.6561	0.6759	0.0893
	LHb	0.7910	0.7779	0.7903	0.8102	0.8019	0.7943	0.0123
	ZI	0.8091	0.8137	0.8178	0.8309	0.8265	0.8196	0.0090
	SPF	0.8261	0.8296	0.8126	0.8114	0.8151	0.8190	0.0083

**Position of seed voxel**	**Seed voxel**	**Pearson correlation values**					**Mean correlation**	**Standard Deviation**
	DLG	0.8280	0.8096	0.8282	0.7246	0.8200	0.8021	0.0440
	PG	0.7521	0.7393	0.7565	0.7639	0.7396	0.7503	0.0107
	MGN	0.8297	0.8611	0.8560	0.8420	0.8018	0.8381	0.0237
	VPL/VPM	0.7955	0.7702	0.7888	0.7874	0.8248	0.7933	0.0199
	VL/VA	0.8722	0.8458	0.8574	0.8673	0.8107	0.8507	0.0245
	VM	0.8899	0.8725	0.8638	0.8122	0.8195	0.8516	0.0340
	MD	0.8851	0.8868	0.8904	0.8998	0.9154	0.8955	0.0125
	LD	0.8347	0.8219	0.8239	0.8473	0.7839	0.8223	0.0237
	Po	0.8665	0.8346	0.8459	0.8781	0.8591	0.8568	0.0171
	AD	0.8337	0.8810	0.8810	0.8480	0.8176	0.8523	0.0284
map.org/agea?seed=P	**AM**	0.9735	0.9655	0.8946	0.9540	0.9132	0.9402	0.0345
	AV	0.8529	0.9053	0.9290	0.8744	0.9398	0.9003	0.0365
	LP	0.8270	0.8067	0.8399	0.8672	0.8780	0.8438	0.0291
	PV	0.7870	0.8447	0.8557	0.8416	0.8389	0.8336	0.0268
	PT	0.9040	0.8763	0.8447	0.8709	0.8457	0.8683	0.0246
	IMD	0.7591	0.7863	0.8035	0.8226	0.8672	0.8077	0.0406
	Re	0.8664	0.8464	0.8552	0.8489	0.8629	0.8560	0.0086
	CM	0.8907	0.9098	0.9098	0.8888	0.8833	0.8965	0.0125
	PF	0.7937	0.7919	0.8065	0.7772	0.7779	0.7894	0.0122
	RT	0.7757	0.8005	0.7339	0.7707	0.7397	0.7641	0.0274
	MHb	0.6128	0.6410	0.6651	0.6278	0.5983	0.6290	0.0258
	LHb	0.8065	0.8124	0.7420	0.8065	0.7419	0.7819	0.0365
	ZI	0.7596	0.7580	0.7797	0.7685	0.7340	0.7600	0.0169
	SPF	0.7866	0.7934	0.8235	0.7939	0.7939	0.7983	0.0144

**Position of seed voxel**	**Seed voxel**	**Pearson correlation values**					**Mean correlation**	**Standard Deviation**
	DLG	0.7359	0.7786	0.7229	0.8288	0.8170	0.7766	0.0472
	PG	0.8166	0.8090	0.7844	0.7951	0.7869	0.7984	0.0140
	MGN	0.7962	0.7335	0.7805	0.7425	0.8279	0.7761	0.0389
	VPL/VPM	0.8342	0.8252	0.8378	0.8075	0.8107	0.8231	0.0136
	VL/VA	0.8635	0.8752	0.8619	0.8645	0.8169	0.8564	0.0227
	VM	0.8241	0.8266	0.8371	0.8478	0.8268	0.8325	0.0099
	MD	0.8413	0.8414	0.8722	0.8673	0.8587	0.8562	0.0144
	LD	0.8472	0.8486	0.8293	0.8240	0.7954	0.8289	0.0216
	Po	0.8143	0.8311	0.8163	0.8286	0.8428	0.8266	0.0117
	AD	0.8786	0.8786	0.8692	0.8380	0.8706	0.8670	0.0168
	AM	0.8607	0.9523	0.9385	0.8858	0.8830	0.9041	0.0393
map.org/agea?seed=P	**AV**	0.8744	0.9764	0.9613	0.9339	0.9074	0.9307	0.0411
	LP	0.7951	0.8514	0.8514	0.8065	0.8089	0.8227	0.0267
	PV	0.7641	0.7874	0.8347	0.8451	0.8140	0.8091	0.0334
	PT	0.7850	0.7795	0.8581	0.8582	0.8118	0.8185	0.0382
	IMD	0.8261	0.8447	0.8518	0.8263	0.8369	0.8372	0.0113
	Re	0.8517	0.8398	0.8258	0.8026	0.8116	0.8263	0.0200
	CM	0.8421	0.8584	0.8571	0.8424	0.8249	0.8450	0.0136
	PF	0.7735	0.7973	0.7912	0.8035	0.8089	0.7949	0.0137
	RT	0.8074	0.8511	0.8107	0.8138	0.7403	0.8047	0.0401
	MHb	0.6283	0.6670	0.6753	0.6452	0.6386	0.6509	0.0197
	LHb	0.7474	0.7955	0.8152	0.7467	0.7467	0.7703	0.0327
	ZI	0.7979	0.8032	0.7785	0.7953	0.8022	0.7954	0.0100
	SPF	0.8220	0.7738	0.7971	0.8121	0.7983	0.8007	0.0182

**Position of seed voxel**	**Seed voxel**	**Pearson correlation values**					**Mean correlation**	**Standard Deviation**
	DLG	0.9206	0.9070	0.9183	0.9034	0.8395	0.8978	0.0334
	PG	0.7261	0.7261	0.7623	0.7690	0.7846	0.7536	0.0264
	MGN	0.9267	0.9245	0.9149	0.9362	0.9015	0.9208	0.0132
	VPL/VPM	0.8897	0.8841	0.8702	0.8770	0.8850	0.8812	0.0076

	VL/VA	0.8805	0.8754	0.8926	0.8794	0.7963	0.8648	0.0388
	VM	0.9083	0.9053	0.8866	0.8947	0.8994	0.8989	0.0086
	MD	0.9245	0.9330	0.8802	0.9282	0.9302	0.9192	0.0220
	LD	0.9051	0.8965	0.9201	0.8974	0.8868	0.9012	0.0124
	Po	0.9463	0.9547	0.9676	0.9542	0.9787	0.9603	0.0128
	AD	0.7828	0.8088	0.7828	0.8391	0.7938	0.8015	0.0236
	AM	0.8788	0.8829	0.8950	0.8925	0.9018	0.8902	0.0093
	AV	0.8612	0.8899	0.9165	0.9152	0.9007	0.8967	0.0227
map.org/agea?seed=P	**LP**	0.9881	0.9900	1.0000	0.9724	0.9759	0.9853	0.0112
	PV	0.8260	0.8163	0.8256	0.8312	0.8696	0.8337	0.0208
	PT	0.7902	0.8457	0.8461	0.8461	0.8457	0.8348	0.0249
	IMD	0.8977	0.9067	0.9025	0.8773	0.9032	0.8975	0.0117
	Re	0.8287	0.8623	0.8287	0.8563	0.8291	0.8410	0.0168
	CM	0.8627	0.8989	0.9093	0.9035	0.9208	0.8990	0.0219
	PF	0.8405	0.8481	0.8007	0.8143	0.8043	0.8216	0.0215
	RT	0.7519	0.7472	0.7644	0.7975	0.8000	0.7722	0.0251
	MHb	0.4715	0.5271	0.5622	0.5815	0.5271	0.5339	0.0420
	LHb	0.7319	0.7526	0.7577	0.7532	0.8379	0.7667	0.0411
	ZI	0.7613	0.8034	0.7760	0.8032	0.7550	0.7798	0.0228
	SPF	0.8239	0.9105	0.8695	0.8837	0.8837	0.8743	0.0318

**Position of seed voxel**	**Seed voxel**	**Pearson correlation values**					**Mean correlation**	**Standard Deviation**
	DLG	0.7927	0.7775	0.8068	0.7821	0.7775	0.7873	0.0125
	PG	0.8169	0.8341	0.8452	0.8169	0.8099	0.8246	0.0146
	MGN	0.8030	0.7828	0.8115	0.8395	0.7485	0.7971	0.0339
	VPL/VPM	0.7715	0.7747	0.7579	0.7696	0.7491	0.7646	0.0107
	VL/VA	0.8178	0.8320	0.8320	0.8289	0.8405	0.8302	0.0082
	VM	0.8842	0.8679	0.8445	0.8330	0.8452	0.8550	0.0207
	MD	0.8579	0.8404	0.8662	0.8622	0.8567	0.8567	0.0098
	LD	0.8386	0.8309	0.8089	0.8101	0.8044	0.8186	0.0152
	Po	0.7903	0.8033	0.8643	0.7944	0.8077	0.8120	0.0300
	AD	0.8243	0.8194	0.8243	0.7975	0.8001	0.8131	0.0133
	AM	0.8760	0.8658	0.8384	0.8156	0.8087	0.8409	0.0297
	AV	0.7914	0.8081	0.8087	0.8004	0.7955	0.8008	0.0076
	LP	0.8903	0.8316	0.8622	0.8567	0.8343	0.8550	0.0239
map.org/agea?seed=P	**PV**	0.8475	0.8680	0.9401	0.9691	0.9195	0.9088	0.0504
	PT	0.8869	0.8893	0.8912	0.8920	0.8510	0.8821	0.0175
	IMD	0.8851	0.9013	0.9273	0.9183	0.9121	0.9088	0.0163
	Re	0.9010	0.8957	0.8903	0.8906	0.9011	0.8957	0.0053
	CM	0.8966	0.9019	0.8592	0.8384	0.8410	0.8674	0.0302
	PF	0.8842	0.8618	0.8464	0.8814	0.8618	0.8671	0.0157
	RT	0.7523	0.7744	0.7934	0.7424	0.7708	0.7667	0.0199
	MHb	0.7723	0.7911	0.7109	0.7825	0.7402	0.7594	0.0333
	LHb	0.8842	0.9232	0.8674	0.8674	0.9165	0.8917	0.0267
	ZI	0.8327	0.8016	0.7941	0.8528	0.8442	0.8251	0.0260
	SPF	0.9006	0.8862	0.8909	0.8745	0.8584	0.8821	0.0162

**Position of seed voxel**	**Seed voxel**	**Pearson correlation values**					**Mean correlation**	**Standard Deviation**
	DLG	0.8151	0.8260	0.8243	0.8336	0.8151	0.8228	0.0079
	PG	0.8321	0.8314	0.8162	0.8367	0.8162	0.8265	0.0096
	MGN	0.7909	0.8650	0.8210	0.7960	0.8421	0.8230	0.0312
	VPL/VPM	0.7991	0.8000	0.8095	0.8163	0.8035	0.8057	0.0072
	VM/VAL	0.8733	0.8379	0.8424	0.8632	0.8512	0.8536	0.0146
	VM	0.8973	0.8665	0.8671	0.8509	0.8680	0.8700	0.0168
	MD	0.8749	0.8698	0.8983	0.8747	0.9193	0.8874	0.0210
	LD	0.8796	0.8631	0.8620	0.8616	0.8959	0.8724	0.0151
	Po	0.8423	0.8508	0.8423	0.8508	0.8624	0.8497	0.0083
	AD	0.8836	0.8691	0.8405	0.8482	0.8836	0.8650	0.0199
	AM	0.8727	0.8938	0.8788	0.8610	0.8685	0.8750	0.0124
	AV	0.8612	0.8574	0.8587	0.8557	0.8612	0.8588	0.0024
	LP	0.8675	0.8784	0.8784	0.8675	0.8747	0.8733	0.0055
	PV	0.9233	0.9181	0.8763	0.8701	0.8515	0.8879	0.0314
map.org/agea?seed=P	**PT**	0.9919	1.0000	0.9577	1.0000	1.0000	0.9899	0.0183
	IMD	0.9170	0.9121	0.9032	0.9121	0.9054	0.9100	0.0056
	Re	0.9395	0.9437	0.9311	0.9358	0.9027	0.9306	0.0163

	CM	0.9088	0.9027	0.8961	0.9203	0.9053	0.9066	0.0089
	PF	0.8476	0.8555	0.8642	0.8476	0.8724	0.8575	0.0108
	RT	0.8222	0.8526	0.7878	0.8119	0.7677	0.8084	0.0325
	MHb	0.5275	0.6010	0.6302	0.6466	0.6083	0.6027	0.0458
	LHb	0.7473	0.8109	0.8379	0.8216	0.8267	0.8089	0.0358
	ZI	0.8170	0.8052	0.8197	0.7975	0.7849	0.8049	0.0143
	SPF	0.8412	0.8378	0.8465	0.8371	0.8389	0.8403	0.0038

Position of seed voxel	Seed voxel	Pearson correlation values					Mean correlation	Standard Deviation
	DLG	0.8847	0.8751	0.8735	0.8847	0.8689	0.8774	0.0071
	PG	0.8080	0.7365	0.7939	0.7854	0.8023	0.7852	0.0285
	MGN	0.8739	0.8411	0.8411	0.8197	0.8342	0.8420	0.0199
	VPL/VPM	0.8732	0.8625	0.8734	0.8593	0.8579	0.8653	0.0075
	VM/VAL	0.8523	0.9215	0.9118	0.8920	0.9106	0.8976	0.0275
	VM	0.9139	0.9094	0.9225	0.8929	0.9079	0.9093	0.0108
	MD	0.9784	0.9784	0.9683	0.9917	0.9753	0.9784	0.0085
	LD	0.9034	0.8879	0.8758	0.8710	0.8859	0.8848	0.0125
	Po	0.9157	0.9169	0.8855	0.9221	0.9179	0.9116	0.0148
	AD	0.8527	0.8252	0.8715	0.8527	0.8252	0.8455	0.0200
	AM	0.9028	0.8872	0.9095	0.9228	0.9004	0.9045	0.0130
	AV	0.8649	0.9227	0.8979	0.8906	0.8990	0.8950	0.0207
	LP	0.8954	0.8820	0.9229	0.8976	0.9047	0.9005	0.0150
	PV	0.7703	0.8054	0.7573	0.8917	0.8642	0.8178	0.0585
	PT	0.8436	0.8362	0.8436	0.9146	0.9068	0.8690	0.0383
map.org/agea?seed=P	**IMD**	0.9937	0.9828	0.9524	0.9077	0.8328	0.9339	0.0656
	Re	0.8811	0.8928	0.9050	0.8811	0.9013	0.8923	0.0111
	CM	0.9629	0.9562	0.9747	0.9851	0.9670	0.9692	0.0111
	PF	0.8227	0.8363	0.8224	0.8307	0.8224	0.8269	0.0063
	RT	0.7702	0.7957	0.7651	0.7889	0.8136	0.7867	0.0197
	MHb	0.4966	0.5663	0.6018	0.6176	0.5689	0.5702	0.0466
	LHb	0.7916	0.7882	0.7882	0.7251	0.7849	0.7756	0.0283
	ZI	0.8028	0.7654	0.7598	0.7504	0.7535	0.7664	0.0212
	SPF	0.8307	0.8503	0.8856	0.8484	0.8430	0.8516	0.0205

Position of seed voxel	Seed voxel	Pearson correlation values					Mean correlation	Standard Deviation
	DLG	0.8417	0.8345	0.8398	0.8318	0.8336	0.8363	0.0042
	PG	0.8105	0.8112	0.7829	0.8005	0.7843	0.7979	0.0137
	MGN	0.8515	0.8602	0.8307	0.8322	0.8387	0.8427	0.0128
	VPL/VPM	0.8603	0.8694	0.8266	0.8191	0.8231	0.8397	0.0233
	VM/VAL	0.8332	0.8761	0.8611	0.8485	0.8261	0.8490	0.0203
	VM	0.9638	0.9289	0.8955	0.9010	0.9193	0.9217	0.0271
	MD	0.9310	0.9296	0.9268	0.8950	0.8865	0.9138	0.0213
	LD	0.8880	0.8617	0.8784	0.8616	0.8395	0.8658	0.0186
	Po	0.8750	0.8717	0.8754	0.8412	0.8643	0.8655	0.0143
	AD	0.7860	0.8091	0.8091	0.8448	0.8219	0.8142	0.0215
	AM	0.8692	0.8965	0.8920	0.8740	0.9059	0.8875	0.0155
	AV	0.8266	0.8459	0.8765	0.8795	0.7488	0.8355	0.0532
	LP	0.8967	0.8776	0.8529	0.8621	0.8776	0.8734	0.0168
	PV	0.8207	0.8390	0.8541	0.9173	0.8769	0.8616	0.0373
	PT	0.8545	0.8756	0.8573	0.8706	0.8545	0.8625	0.0099
	IMD	0.9077	0.9025	0.9108	0.8988	0.9038	0.9047	0.0047
map.org/agea?seed=P	**Re**	0.8998	0.9517	0.9618	0.9419	0.9314	0.9373	0.0238
	CM	0.9042	0.9020	0.9020	0.9083	0.9293	0.9092	0.0115
	PF	0.8598	0.8466	0.8414	0.8557	0.8466	0.8500	0.0075
	RT	0.7460	0.7863	0.7540	0.7888	0.7306	0.7611	0.0255
	MHb	0.6530	0.7070	0.7323	0.7042	0.7173	0.7028	0.0299
	LHb	0.8814	0.8525	0.8051	0.7800	0.8795	0.8397	0.0454
	ZI	0.8818	0.8715	0.8735	0.8159	0.7547	0.8395	0.0541
	SPF	0.8633	0.8840	0.8758	0.8597	0.8864	0.8738	0.0120

Position of seed voxel	Seed voxel	Pearson correlation values					Mean correlation	Standard Deviation
	DLG	0.8642	0.8749	0.8744	0.8769	0.8826	0.8746	0.0067
	PG	0.7698	0.8080	0.7854	0.7857	0.7939	0.7886	0.0139
	MGN	0.8460	0.8473	0.8674	0.8791	0.7514	0.8382	0.0505

	VPL/VPM	0.8732	0.8668	0.8782	0.8850	0.8699	0.8746	0.0072
	VM/VAL	0.8810	0.9106	0.9068	0.8842	0.8920	0.8949	0.0133
	VM	0.9094	0.9225	0.9164	0.9252	0.9036	0.9154	0.0090
	MD	0.9784	0.9784	0.9651	0.9917	0.9683	0.9764	0.0104
	LD	0.8752	0.8965	0.8820	0.8879	0.8758	0.8835	0.0089
	Po	0.8844	0.8855	0.9169	0.9221	0.9179	0.9054	0.0187
	AD	0.8252	0.8166	0.8527	0.7503	0.8252	0.8140	0.0381
	AM	0.9037	0.9028	0.9194	0.9281	0.9194	0.9147	0.0110
	AV	0.8774	0.8653	0.8730	0.8939	0.8990	0.8817	0.0142
	LP	0.8900	0.8976	0.8820	0.8954	0.9030	0.8936	0.0080
	PV	0.7703	0.7545	0.7573	0.8762	0.8811	0.8079	0.0649
	PT	0.8436	0.8362	0.8958	0.8958	0.9068	0.8756	0.0330
	IMD	0.9503	0.9766	0.9868	1.0000	0.9714	0.9770	0.0185
	Re	0.8811	0.8928	0.9156	0.9142	0.9013	0.9010	0.0146
map.org/agea?seed=P	**CM**	1.0000	0.9670	0.9660	0.9851	0.9747	0.9786	0.0142
	PF	0.8728	0.8700	0.8769	0.8307	0.8224	0.8546	0.0259
	RT	0.8498	0.8389	0.7789	0.8046	0.8230	0.8190	0.0282
	MHb	0.4966	0.5663	0.6018	0.6176	0.6686	0.5902	0.0640
	LHb	0.8474	0.8708	0.8686	0.8507	0.7936	0.8462	0.0312
	ZI	0.7801	0.8028	0.7705	0.7542	0.7634	0.7742	0.0186
	SPF	0.8176	0.8172	0.8173	0.8477	0.8225	0.8245	0.0132

Position of seed voxel	Seed voxel	Pearson correlation values					Mean correlation	Standard Deviation
	DLG	0.8005	0.8426	0.8226	0.8348	0.8129	0.8227	0.0168
	PG	0.8519	0.8600	0.8426	0.7245	0.8599	0.8278	0.0582
	MGN	0.7228	0.8563	0.6053	0.7709	0.7704	0.7451	0.0918
	VPL/VPM	0.7969	0.8186	0.7910	0.7863	0.7915	0.7969	0.0127
	VM/VAL	0.8405	0.8264	0.8092	0.8097	0.8182	0.8208	0.0131
	VM	0.8471	0.8599	0.8343	0.8803	0.8561	0.8555	0.0170
	MD	0.8173	0.8427	0.8390	0.8547	0.8308	0.8369	0.0139
	LD	0.8245	0.8348	0.8329	0.8095	0.8080	0.8219	0.0127
	Po	0.8415	0.8518	0.8397	0.8071	0.7940	0.8268	0.0249
	AD	0.7853	0.7578	0.8318	0.8318	0.7850	0.7983	0.0325
	AM	0.7833	0.7924	0.8137	0.8043	0.7998	0.7987	0.0116
	AV	0.7633	0.7975	0.7529	0.7850	0.8079	0.7813	0.0230
	LP	0.8620	0.8389	0.7924	0.8014	0.8209	0.8231	0.0282
	PV	0.7575	0.7440	0.7336	0.7989	0.8474	0.7763	0.0469
	PT	0.7779	0.7979	0.8136	0.8074	0.7806	0.7955	0.0159
	IMD	0.8299	0.8257	0.8389	0.8426	0.8561	0.8386	0.0119
	Re	0.8025	0.8409	0.8559	0.8609	0.8441	0.8409	0.0230
	CM	0.8385	0.8488	0.8457	0.8557	0.8860	0.8549	0.0184
map.org/agea?seed=P	**PF**	0.9393	0.9460	0.9815	0.9858	0.9650	0.9635	0.0207
	RT	0.7455	0.7951	0.7323	0.8009	0.7723	0.7692	0.0300
	MHb	0.6498	0.7005	0.7334	0.6899	0.6828	0.6913	0.0302
	LHb	0.8747	0.8995	0.8655	0.7979	0.7664	0.8408	0.0561
	ZI	0.8314	0.8479	0.8627	0.8454	0.8033	0.8381	0.0224
	SPF	0.8984	0.9265	0.8901	0.8854	0.8594	0.8920	0.0242

Position of seed voxel	Seed voxel	Pearson correlation values					Mean correlation	Standard Deviation
	DLG	0.7946	0.7876	0.8093	0.8162	0.8298	0.8075	0.0169
	PG	0.8789	0.8730	0.8857	0.8600	0.8723	0.8740	0.0095
	MGN	0.7322	0.7077	0.7730	0.7223	0.7960	0.7462	0.0369
	VPL/VPM	0.8216	0.8013	0.8185	0.8401	0.8206	0.8204	0.0138
	VM/VAL	0.8128	0.8342	0.8498	0.8416	0.8325	0.8342	0.0138
	VM	0.8087	0.7833	0.8572	0.8098	0.8461	0.8210	0.0302
	MD	0.8046	0.8122	0.8222	0.8106	0.8188	0.8137	0.0069
	LD	0.8250	0.8308	0.8033	0.8198	0.8120	0.8182	0.0108
	Po	0.7873	0.7682	0.7833	0.7717	0.7898	0.7801	0.0096
	AD	0.8160	0.8090	0.8160	0.8126	0.8126	0.8132	0.0029
	AM	0.7699	0.8264	0.7742	0.7593	0.7972	0.7854	0.0268
	AV	0.7753	0.8646	0.8230	0.7973	0.8230	0.8166	0.0334
	LP	0.7892	0.7775	0.7764	0.7786	0.7764	0.7796	0.0054
	PV	0.7538	0.7621	0.7566	0.7423	0.7266	0.7483	0.0141
	PT	0.7687	0.7734	0.7734	0.7814	0.7565	0.7707	0.0091
	IMD	0.7628	0.7628	0.7740	0.7776	0.7682	0.7691	0.0066

	Re	0.7672	0.7483	0.7571	0.7799	0.7847	0.7674	0.0152
	CM	0.7781	0.7662	0.7651	0.7738	0.7781	0.7723	0.0063
	PF	0.7961	0.7991	0.7911	0.7704	0.7880	0.7889	0.0112
map.org/agea?seed=P	**RT**	0.8896	0.9507	0.9600	0.9918	0.9234	0.9431	0.0386
	MHb	0.5693	0.6106	0.6130	0.6320	0.6710	0.6192	0.0369
	LHb	0.8384	0.8422	0.8273	0.8213	0.8248	0.8308	0.0090
	ZI	0.8753	0.8848	0.8698	0.8769	0.8807	0.8775	0.0057
	SPF	0.7650	0.7876	0.7962	0.8182	0.8264	0.7987	0.0246

Position of seed voxel	Seed voxel	Pearson correlation values					Mean correlation	Standard Deviation
	DLG	0.5584	0.5788	0.5648	0.5692	0.5488	0.5640	0.0113
	PG	0.6431	0.6428	0.6324	0.6436	0.6276	0.6379	0.0074
	MGN	0.5503	0.6123	0.5318	0.5467	0.5141	0.5510	0.0371
	VPL/VPM	0.5554	0.5553	0.5387	0.5443	0.5628	0.5513	0.0097
	VM/VAL	0.5731	0.5933	0.6129	0.5718	0.5686	0.5839	0.0189
	VM	0.6584	0.5712	0.5886	0.6073	0.5780	0.6007	0.0350
	MD	0.5961	0.5865	0.5787	0.5766	0.5806	0.5837	0.0079
	LD	0.6061	0.6116	0.6148	0.5944	0.6122	0.6078	0.0081
	Po	0.5916	0.5446	0.6261	0.6173	0.5516	0.5862	0.0371
	AD	0.6289	0.6162	0.6419	0.6617	0.6289	0.6355	0.0172
	AM	0.5830	0.5519	0.5538	0.5554	0.5538	0.5596	0.0132
	AV	0.6092	0.5817	0.6092	0.5563	0.5763	0.5865	0.0227
	LP	0.5996	0.5712	0.6350	0.5625	0.5468	0.5830	0.0348
	PV	0.6634	0.6782	0.6733	0.7071	0.7112	0.6866	0.0213
	PT	0.6302	0.6375	0.6302	0.6361	0.6302	0.6328	0.0036
	IMD	0.6050	0.6252	0.5991	0.5904	0.6130	0.6065	0.0133
	Re	0.6266	0.6277	0.6398	0.6196	0.6256	0.6279	0.0074
	CM	0.6077	0.6003	0.5934	0.5798	0.5854	0.5933	0.0112
	PF	0.6157	0.6429	0.6550	0.6353	0.6429	0.6384	0.0145
	RT	0.6081	0.6152	0.6130	0.6072	0.6023	0.6092	0.0051
map.org/agea?seed=P	**MHb**	0.8147	0.9575	0.9020	0.9723	0.9249	0.9143	0.0621
	LHb	0.7897	0.7732	0.6994	0.7283	0.7305	0.7442	0.0366
	ZI	0.6171	0.6154	0.6093	0.6135	0.6200	0.6151	0.0040
	SPF	0.7167	0.6700	0.6488	0.6531	0.6726	0.6722	0.0269

Position of seed voxel	Seed voxel	Pearson correlation values					Mean correlation	Standard Deviation
	DLG	0.7714	0.7603	0.7418	0.7731	0.7463	0.7586	0.0142
	PG	0.8167	0.8125	0.8259	0.8223	0.8228	0.8200	0.0054
	MGN	0.7416	0.8057	0.8057	0.8490	0.7185	0.7841	0.0530
	VPL/VPM	0.7939	0.7703	0.7490	0.7376	0.7483	0.7598	0.0224
	VM/VAL	0.7978	0.7890	0.7601	0.7650	0.7564	0.7737	0.0185
	VM	0.7744	0.8735	0.8019	0.8337	0.8040	0.8175	0.0377
	MD	0.7792	0.7631	0.7652	0.7689	0.7630	0.7679	0.0068
	LD	0.7982	0.8007	0.8007	0.7789	0.7989	0.7955	0.0093
	Po	0.8323	0.7901	0.8034	0.7620	0.7466	0.7869	0.0339
	AD	0.7864	0.7987	0.7932	0.8056	0.8056	0.7979	0.0083
	AM	0.7919	0.7693	0.8293	0.7315	0.7331	0.7710	0.0413
	AV	0.7388	0.7900	0.7790	0.7376	0.7746	0.7640	0.0242
	LP	0.8348	0.8011	0.7870	0.8348	0.8523	0.8220	0.0270
	PV	0.7868	0.7789	0.7870	0.8791	0.8342	0.8132	0.0428
	PT	0.8450	0.8321	0.8216	0.8216	0.8240	0.8289	0.0100
	IMD	0.7849	0.7890	0.7952	0.8056	0.7946	0.7939	0.0078
	Re	0.8459	0.8215	0.8198	0.8245	0.7862	0.8196	0.0214
	CM	0.7934	0.7776	0.8319	0.8151	0.8298	0.8096	0.0236
	PF	0.8275	0.7935	0.8615	0.8123	0.8456	0.8281	0.0268
	RT	0.7864	0.8113	0.7846	0.8099	0.7675	0.7919	0.0186
	MHb	0.8074	0.8629	0.7732	0.8015	0.8227	0.8135	0.0329
map.org/agea?seed=P	**LHb**	0.9067	0.9194	0.9434	1.0000	0.9634	0.9466	0.0370
	ZI	0.8298	0.8385	0.8264	0.8237	0.8404	0.8318	0.0074
	SPF	0.8302	0.8005	0.8334	0.8190	0.8140	0.8194	0.0132

Position of seed voxel	Seed voxel	Pearson correlation values					Mean correlation	Standard Deviation
	DLG	0.7854	0.7748	0.7820	0.7913	0.7800	0.7827	0.0062
	PG	0.9480	0.9032	0.8715	0.9320	0.8448	0.8999	0.0424

	MGN	0.7591	0.8305	0.7162	0.7016	0.7135	0.7442	0.0529
	VPL/VPM	0.7812	0.7797	0.7805	0.7830	0.7747	0.7798	0.0031
	VM/VAL	0.7659	0.7372	0.7852	0.7722	0.7752	0.7671	0.0181
	VM	0.7813	0.7633	0.7396	0.7760	0.7657	0.7652	0.0161
	MD	0.7687	0.7810	0.7720	0.7683	0.7671	0.7714	0.0057
	LD	0.7826	0.8027	0.7943	0.7987	0.7925	0.7942	0.0076
	Po	0.8172	0.7552	0.7932	0.7617	0.8146	0.7884	0.0290
	AD	0.8201	0.8109	0.8201	0.8097	0.8055	0.8133	0.0066
	AM	0.7579	0.7608	0.7330	0.7350	0.7741	0.7522	0.0177
	AV	0.7925	0.7483	0.7908	0.7483	0.7299	0.7620	0.0281
	LP	0.7724	0.7823	0.7630	0.7837	0.7661	0.7735	0.0093
	PV	0.7741	0.7707	0.7710	0.8231	0.7875	0.7853	0.0222
	PT	0.7941	0.8053	0.8069	0.7966	0.7749	0.7956	0.0128
	IMD	0.7669	0.7705	0.7666	0.7687	0.7725	0.7690	0.0025
	Re	0.7828	0.7798	0.7895	0.7802	0.7895	0.7844	0.0048
	CM	0.7722	0.7740	0.7722	0.7776	0.7755	0.7743	0.0023
	PF	0.8353	0.8445	0.8732	0.8605	0.8409	0.8509	0.0156
	RT	0.8346	0.8680	0.8702	0.8638	0.8498	0.8573	0.0150
	MHb	0.5430	0.5986	0.6187	0.6372	0.6510	0.6097	0.0422
	LHb	0.8192	0.8349	0.8544	0.8722	0.8296	0.8421	0.0212
map.org/agea?seed=P	ZI	0.9417	0.9731	0.8983	0.9199	0.9651	0.9396	0.0311
	**SPF**	0.8765	0.8992	0.8971	0.9030	0.9220	0.8996	0.0162

**Position of seed voxel**	**Seed voxel**	**Pearson correlation values**					**Mean correlation**	**Standard Deviation**
	DLG	0.8341	0.8278	0.8692	0.8602	0.8615	0.8506	0.0184
	PG	0.8643	0.9125	0.9151	0.8451	0.9125	0.8899	0.0329
	MGN	0.8497	0.8642	0.8309	0.8725	0.8295	0.8494	0.0193
	VPL/VPM	0.8213	0.8034	0.7997	0.8203	0.8306	0.8151	0.0130
	VM/VAL	0.8491	0.8626	0.8605	0.8059	0.8216	0.8399	0.0251
	VM	0.8722	0.8730	0.9037	0.9238	0.8828	0.8911	0.0223
	MD	0.8477	0.8516	0.8570	0.8864	0.8543	0.8594	0.0155
	LD	0.8103	0.8381	0.8291	0.8335	0.8426	0.8307	0.0125
	Po	0.8757	0.8371	0.8436	0.8302	0.8429	0.8459	0.0175
	AD	0.7942	0.8144	0.7986	0.8111	0.7986	0.8034	0.0088
	AM	0.8467	0.8107	0.8317	0.8457	0.8256	0.8321	0.0150
	AV	0.8269	0.8338	0.7867	0.8121	0.8063	0.8132	0.0185
	LP	0.8837	0.8406	0.9007	0.9333	0.8939	0.8904	0.0335
	PV	0.8480	0.7562	0.8186	0.8073	0.8363	0.8133	0.0356
	PT	0.8404	0.8438	0.8465	0.8334	0.8347	0.8398	0.0057
	IMD	0.8323	0.8323	0.8387	0.8479	0.8484	0.8399	0.0080
	Re	0.8777	0.8960	0.8671	0.8703	0.8642	0.8751	0.0127
	CM	0.8505	0.8439	0.8606	0.8533	0.8592	0.8535	0.0068
	PF	0.8770	0.8866	0.8939	0.8801	0.8766	0.8828	0.0074
	RT	0.8793	0.8562	0.8210	0.7818	0.7959	0.8268	0.0407
	MHb	0.6668	0.7049	0.7380	0.6919	0.7094	0.7022	0.0260
	LHb	0.8429	0.8190	0.8799	0.8578	0.9135	0.8626	0.0361
	**ZI**	0.9497	0.9096	0.9077	0.9007	0.8976	0.9131	0.0211
map.org/agea?seed=P	SPF	0.9037	0.9512	0.9866	0.9899	0.9745	0.9612	0.0355

Mean correlation

	DLG	PG	MGN	VPL/VPM	VA/VL	VM	MD	LD	Po	AD	AM	AV	LP	PV	PT	IMD	Re	CM	PF	RT	MHb	LHb	ZI	SPF
**DLG**	0.9726	0.8061	0.8993	0.9289	0.9190	0.8836	0.9098	0.9046	0.9026	0.8176	0.8021	0.7766	0.8978	0.7873	0.8228	0.8774	0.8363	0.8746	0.8227	0.8075	0.5640	0.7586	0.7827	0.8506
**PG**	0.7878	0.9785	0.8134	0.7487	0.8177	0.8032	0.7796	0.8038	0.8317	0.7846	0.7503	0.7984	0.7536	0.8246	0.8265	0.7852	0.7979	0.7886	0.8278	0.8740	0.6379	0.8200	0.8999	0.8899
**MGN**	0.8025	0.7603	0.9571	0.8499	0.7997	0.8057	0.9027	0.8269	0.9067	0.7338	0.8381	0.7761	0.9208	0.7971	0.8230	0.8420	0.8427	0.8382	0.7451	0.7462	0.5510	0.7841	0.7442	0.8494
**VPL/VPM**	0.9295	0.7890	0.8880	0.9867	0.9412	0.8932	0.9100	0.9105	0.8984	0.8302	0.7933	0.8231	0.8812	0.7646	0.8057	0.8653	0.8397	0.8746	0.7969	0.8204	0.5513	0.7598	0.7798	0.8151
**VA/VL**	0.9140	0.7724	0.8858	0.9111	0.9866	0.9230	0.9419	0.9386	0.8897	0.8460	0.8507	0.8564	0.8648	0.8302	0.8536	0.8976	0.8490	0.8949	0.8208	0.8342	0.5839	0.7737	0.7671	0.8399
**VM**	0.8856	0.7952	0.8790	0.8812	0.9188	0.9470	0.9363	0.8890	0.8935	0.8121	0.8516	0.8325	0.8989	0.8550	0.8700	0.9093	0.9217	0.9154	0.8555	0.8210	0.6007	0.8175	0.7652	0.8911
**MD**	0.9013	0.7822	0.9070	0.9172	0.9281	0.9142	0.9765	0.9073	0.9291	0.8223	0.8955	0.8562	0.9192	0.8567	0.8874	0.9784	0.9138	0.9764	0.8369	0.8137	0.5837	0.7679	0.7714	0.8594
**LD**	0.8578	0.8020	0.8652	0.9431	0.9015	0.8952	0.9353	0.9432	0.8824	0.8664	0.8223	0.8289	0.9012	0.8186	0.8724	0.8848	0.8658	0.8835	0.8219	0.8182	0.6078	0.7955	0.7942	0.8307
**Po**	0.8863	0.7921	0.9246	0.8850	0.8906	0.8871	0.9382	0.8910	0.9712	0.7856	0.8568	0.8266	0.9603	0.8120	0.8497	0.9116	0.8655	0.9054	0.8268	0.7801	0.5862	0.7869	0.7884	0.8459
**AD**	0.8335	0.8179	0.7819	0.8439	0.8442	0.8201	0.8633	0.8580	0.8381	0.9594	0.8523	0.8670	0.8015	0.8131	0.8650	0.8455	0.8142	0.8140	0.7983	0.8132	0.6355	0.7979	0.8133	0.8034
**AM**	0.8452	0.7877	0.8549	0.8028	0.8683	0.8744	0.9116	0.8864	0.8907	0.8722	0.9402	0.9041	0.8902	0.8409	0.8750	0.9045	0.8875	0.9147	0.7987	0.7854	0.5596	0.7710	0.7522	0.8321
**AV**	0.8629	0.7811	0.7867	0.8379	0.8634	0.8509	0.8872	0.9056	0.8936	0.8964	0.9003	0.9307	0.8967	0.8008	0.8588	0.8950	0.8355	0.8817	0.7813	0.8166	0.5865	0.7640	0.7620	0.8132
**LP**	0.8694	0.7856	0.9306	0.8521	0.8800	0.8948	0.9177	0.8885	0.9540	0.8190	0.8438	0.8227	0.9853	0.8550	0.8733	0.9005	0.8734	0.8936	0.8231	0.7796	0.5830	0.8220	0.7735	0.8904
**PV**	0.7505	0.8105	0.7697	0.7177	0.8019	0.8006	0.8183	0.7960	0.8531	0.8153	0.8336	0.8091	0.8337	0.9088	0.8879	0.8178	0.8616	0.8079	0.7763	0.7483	0.6866	0.8132	0.7853	0.8133
**PT**	0.7662	0.8212	0.7685	0.7611	0.8001	0.8495	0.8709	0.7979	0.8300	0.8057	0.8683	0.8185	0.8348	0.8821	0.9899	0.8690	0.8625	0.8756	0.7955	0.7707	0.6328	0.8289	0.7956	0.8398
**IMD**	0.8264	0.7975	0.8442	0.8461	0.8720	0.9053	0.9085	0.8457	0.9024	0.8121	0.8077	0.8372	0.8975	0.9088	0.9100	0.9339	0.9047	0.9770	0.8386	0.7691	0.6065	0.7939	0.7690	0.8399
**Re**	0.8099	0.8193	0.8131	0.7579	0.7970	0.8696	0.8701	0.8284	0.8574	0.8279	0.8560	0.8263	0.8410	0.8957	0.9306	0.8923	0.9373	0.9010	0.8409	0.7674	0.6279	0.8196	0.7844	0.8751
**CM**	0.8260	0.8004	0.8688	0.8038	0.8704	0.8912	0.9272	0.8714	0.9218	0.8316	0.8965	0.8450	0.8990	0.8674	0.9066	0.9692	0.9092	0.9786	0.8549	0.7723	0.5933	0.8096	0.7743	0.8535
**PF**	0.7884	0.8418	0.8348	0.7843	0.8001	0.8213	0.8501	0.8604	0.8655	0.8270	0.7894	0.7949	0.8216	0.8671	0.8575	0.8269	0.8500	0.8546	0.9635	0.7889	0.6384	0.8281	0.8509	0.8828
**RT**	0.8344	0.8704	0.7810	0.7976	0.8349	0.8152	0.8383	0.8067	0.8251	0.8374	0.7641	0.8047	0.7722	0.7667	0.8084	0.7867	0.7611	0.8190	0.7692	0.9431	0.6092	0.7919	0.8573	0.8268
**MHb**	0.5863	0.6227	0.5945	0.5405	0.5793	0.6436	0.5853	0.6617	0.6263	0.6759	0.6290	0.6509	0.5339	0.7594	0.6027	0.5702	0.7028	0.5902	0.6913	0.6192	0.9143	0.8135	0.6097	0.7022
**LHb**	0.6970	0.8741	0.6555	0.7226	0.7740	0.7770	0.7935	0.7465	0.7426	0.7943	0.7819	0.7703	0.7667	0.8917	0.8089	0.7756	0.8397	0.8462	0.8408	0.8308	0.7442	0.9466	0.8421	0.8626
**ZI**	0.7846	0.9267	0.8356	0.7465	0.8104	0.8007	0.7914	0.8315	0.8191	0.8196	0.7600	0.7954	0.7798	0.8251	0.8049	0.7664	0.8395	0.7742	0.8381	0.8775	0.6151	0.8318	0.9396	0.9131
**SPF**	0.8119	0.9022	0.8466	0.8142	0.8179	0.8974	0.8454	0.8447	0.8986	0.8190	0.7983	0.8007	0.8743	0.8821	0.8403	0.8516	0.8738	0.8245	0.8920	0.7987	0.6722	0.8194	0.8996	0.9612

Mean correlation

	DLG	PG	MGN	VPL/VP	VA/VL	VM	MD	LD	Po	AD	AM	AV	LP	PV	PT	IMD	Re	CM	PF	RT	MHb	LHb	ZI	SPF
DLG	0.9726																							
PG	0.7969	0.9785																						
MGN	0.8509	0.7868	0.9571																					
VPL/VP	0.9292	0.7689	0.8690	0.9867																				
VA/VL	0.9165	0.7951	0.8428	0.9261	0.9866																			
VM	0.8846	0.7992	0.8424	0.8872	0.9209	0.9470																		
MD	0.9055	0.7809	0.9049	0.9136	0.9350	0.9253	0.9765																	
LD	0.8812	0.8029	0.8461	0.9268	0.9201	0.8921	0.9213	0.9432																
Po	0.8944	0.8119	0.9156	0.8917	0.8902	0.8903	0.9336	0.8867	0.9712															
AD	0.8256	0.8012	0.7578	0.8371	0.8451	0.8161	0.8428	0.8622	0.8119	0.9594														
AM	0.8236	0.7690	0.8465	0.7981	0.8595	0.8630	0.9035	0.8544	0.8738	0.8622	0.9402													
AV	0.8198	0.7898	0.7814	0.8305	0.8599	0.8417	0.8717	0.8672	0.8601	0.8817	0.9022	0.9307												
LP	0.8836	0.7696	0.9257	0.8667	0.8724	0.8968	0.9184	0.8948	0.9571	0.8102	0.8670	0.8597	0.9853											
PV	0.7689	0.8175	0.7834	0.7412	0.8161	0.8278	0.8375	0.8073	0.8326	0.8142	0.8372	0.8049	0.8444	0.9088										
PT	0.7945	0.8239	0.7957	0.7834	0.8269	0.8598	0.8792	0.8352	0.8399	0.8354	0.8716	0.8387	0.8540	0.8850	0.9899									
IMD	0.8519	0.7914	0.8276	0.8557	0.8848	0.9073	0.9435	0.8653	0.9070	0.8288	0.8561	0.8661	0.8990	0.8633	0.8895	0.9339								
Re	0.8231	0.8086	0.8558	0.7988	0.8230	0.8956	0.8919	0.8471	0.8615	0.8211	0.8717	0.8309	0.8572	0.8787	0.8965	0.8985	0.9373							
CM	0.8503	0.7945	0.8535	0.8392	0.8827	0.9033	0.9518	0.8774	0.9136	0.8228	0.9056	0.8634	0.8963	0.8377	0.8911	0.9731	0.9051	0.9786						
PF	0.8056	0.8348	0.7900	0.7906	0.8104	0.8384	0.8435	0.8412	0.8462	0.8127	0.7941	0.7881	0.8224	0.8217	0.8265	0.8328	0.8454	0.8548	0.9635					
RT	0.8210	0.8722	0.7636	0.8090	0.8346	0.8181	0.8260	0.8124	0.8026	0.8253	0.7748	0.8107	0.7759	0.7575	0.7896	0.7779	0.7643	0.7957	0.7791	0.9431				
MHb	0.5751	0.6303	0.6033	0.5459	0.5816	0.6222	0.5845	0.6347	0.6063	0.6557	0.5943	0.6187	0.5585	0.7230	0.6178	0.5884	0.6653	0.5918	0.6648	0.6142	0.9143			
LHb	0.7278	0.8471	0.8099	0.7412	0.7738	0.7972	0.7807	0.7710	0.7647	0.7961	0.7764	0.7672	0.7943	0.8525	0.8189	0.7847	0.8296	0.8279	0.8344	0.8114	0.7789	0.9466		
ZI	0.7837	0.9133	0.7899	0.7632	0.7888	0.7829	0.7814	0.8128	0.8037	0.8164	0.7561	0.7787	0.7766	0.8052	0.8002	0.7677	0.8119	0.7743	0.8445	0.8674	0.6124	0.8369	0.9396	
SPF	0.8509	0.8758	0.8480	0.8146	0.8289	0.8942	0.8524	0.8377	0.8722	0.8112	0.8152	0.8069	0.8824	0.8477	0.8400	0.8458	0.8745	0.8390	0.8874	0.8128	0.6872	0.8410	0.9063	0.9612
