# Supplementary material for: Molecular anatomy of the thalamic complex and the underlying transcription factors
Source: Brain Struct Funct. 2015 May 12;221:2493–510. doi: 10.1007/s00429-015-1052-5 (PMC4884203; doi:10.1007/s00429-015-1052-5)
Supplement: Supplementary file 5 — Supplementary material 5 (PDF 460 kb) [file 429_2015_1052_MOESM5_ESM.pdf]

Supplementary Material

Figure S1

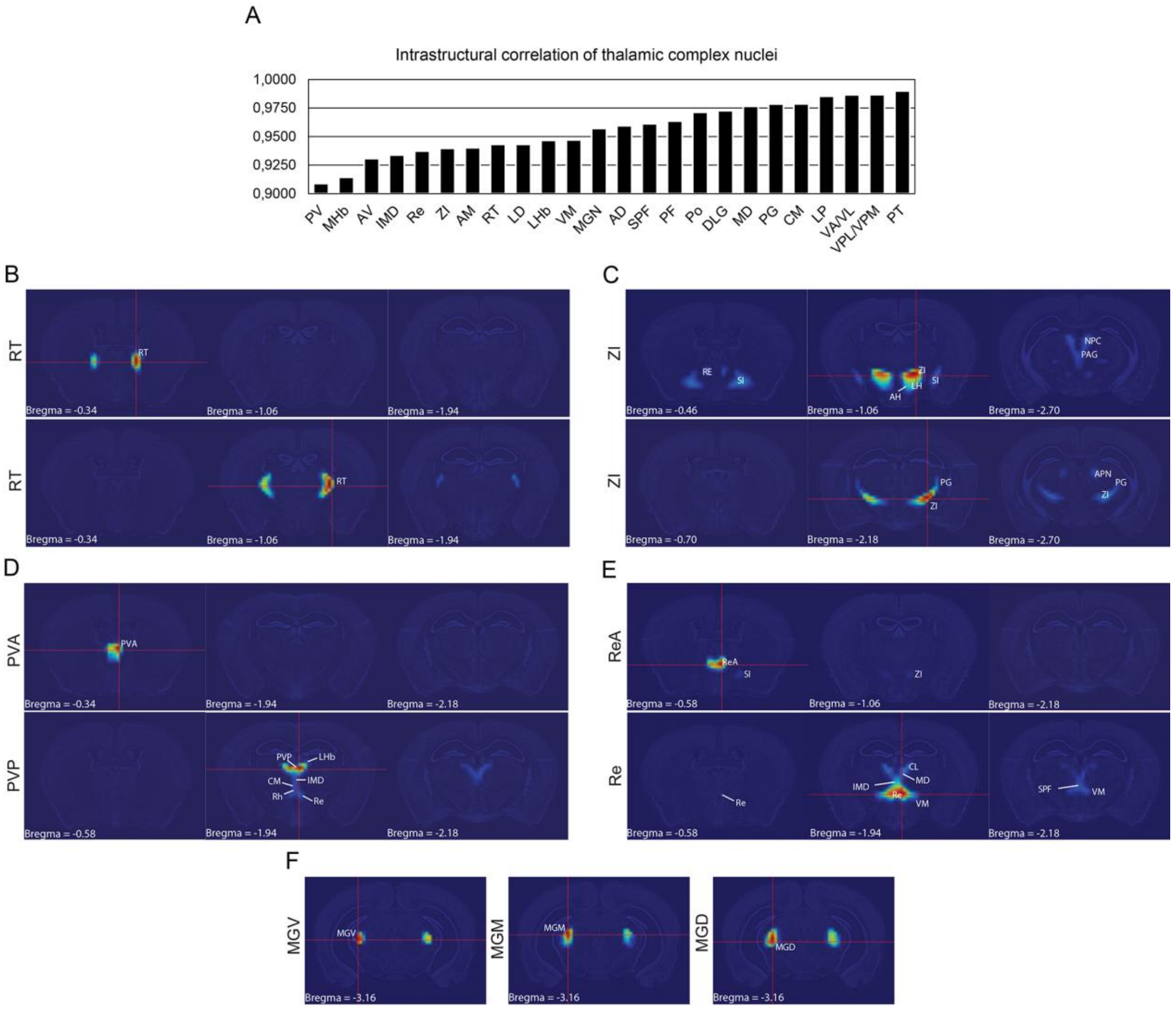

Figure S1. (A) Thalamic complex nuclei ranked by increasing value of intrastructural correlation calculated from AGEA analysis. (B-F) AGEA spatial gene expression correlation maps for selected heterogeneous thalamic nuclei. The computed correlation values are displayed with false-color images using a blue-to-red color scale ("heat map") with the threshold interval set to [0.9, 1]. Nuclei with intrastructural similarity lower than 0.95 (see text) were reexamined by placing additional seed voxels in different parts of them; this revealed molecular subdomains within (A) RT, (B) ZI, (C) PV, (D) Re nuclei. Three panels ordered from rostral to caudal are shown for each seed voxel. The heterogeneity of MGN (E) was reexamined by placing seed voxels in different parts of this nucleus.

Figure S2

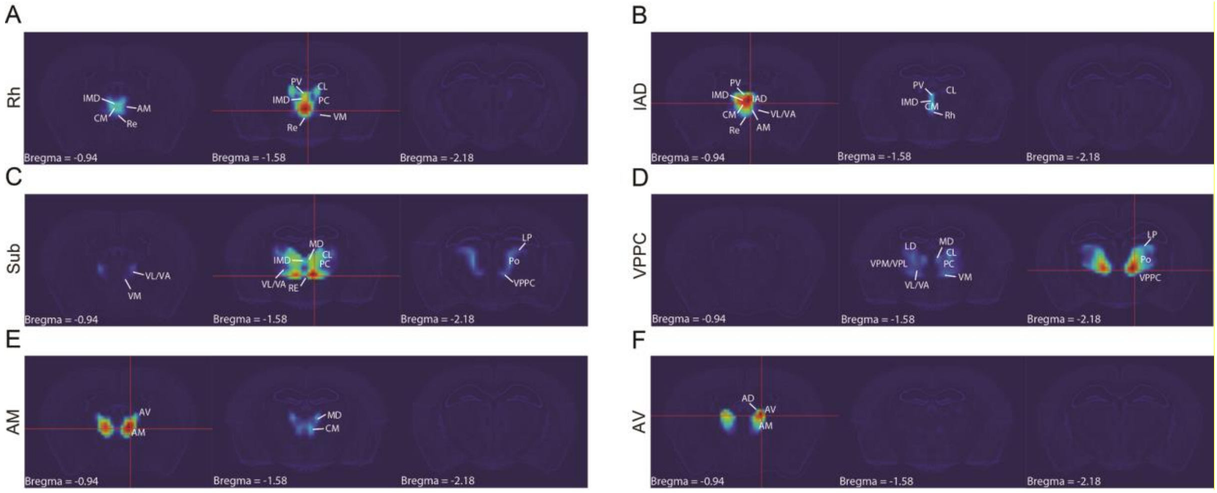

Figure S2. AGEA spatial gene expression correlation maps for additional thalamic nuclei. Seed voxels were placed in (A) Rh, (B) IAD, (C) Sub, (D) VPPC, (E) AM and (F) AV. The computed correlation values are displayed with false-color images using a blue-to-red color scale (“heat map”), with the threshold interval set to [0.9, 1]. For a detailed description of AGEA spatial gene expression correlation maps, see (Ng et al. 2009). Three panels ordered from rostral to caudal are shown for each seed voxel.

Figure S3

Prethalamus

Entrez id	Gene symbol	Gene name
2104	ESRRG	estrogen-related receptor gamma
3170	FOXA2	forkhead box A2
3231	HOXD1	homeobox D1
3670	ISL1	ISL LIM homeobox 1
4212	MEIS2	meis homeobox 2
9603	NFE2L3	nuclear factor (erythroid-derived 2)-like 3
4821	NKX2-2	NK2 homeobox 2
6496	SIX3	SIX homeobox 3
30812	SOX8	SRY (sex determining region Y)-box 8
9839	ZEB2	zinc finger E-box binding homeobox 2

Epithalamus

Entrez id	Gene symbol	Gene name
79190	IRX6	iroquois homeobox 6
4760	NEUROD1	neuronal differentiation 1
4929	NR4A2	nuclear receptor subfamily 4, group A, member 2
5457	POU4F1	POU class 4 homeobox 1
5459	POU4F3	POU class 4 homeobox 3

Thalamus

Entrez id	Gene symbol	Gene name
8553	BHLHE40	basic helix-loop-helix family, member e40
2115	ETV1	ets variant 1
27086	FOXP1	forkhead box P1
93986	FOXP2	forkhead box P2
2625	GATA3	GATA binding protein 3
2637	GBX2	gastrulation brain homeobox 2
3400	ID4	inhibitor of DNA binding 4, dominant negative helix-loop-helix protein
51176	LEF1	lymphoid enhancer-binding factor 1
9355	LHX2	LIM homeobox 2
56956	LHX9	LIM homeobox 9
84504	NKX6-2	NK6 homeobox 2
7025	NR2F1	nuclear receptor subfamily 2, group F, member 1
5015	OTX2	orthodenticle homeobox 2
5629	PROX1	prospero homeobox 1
6095	RORA	RAR-related orphan receptor A
83482	SCRT1	scratch homolog 1, zinc finger protein
6474	SHOX2	short stature homeobox 2
8403	SOX14	SRY (sex determining region Y)-box 14
6934	TCF7L2	transcription factor 7-like 2 (T-cell specific, HMG-box)
9760	TOX	thymocyte selection-associated high mobility group box
463	ZFHX3	zinc finger homeobox 3
7545	ZIC1	zic family member 1
7546	ZIC2	zic family member 2
7547	ZIC3	zic family member 3
84107	ZIC4	zic family member 4
79698	ZMAT4	zinc finger, matrin-type 4
151126	ZNF385B	zinc finger protein 385B
23090	ZNF423	zinc finger protein 423
91752	ZNF804A	zinc finger protein 804a

Figure S3. List of transcription factors enriched in human thalamic complex. Genes were selected after manual inspection of genes found using "Differential Search tool" in Human Brain Atlas (http://human.brain-map.org/). Genes marked in red are conserved between human and mouse.

Supplementary experimental procedures

Sequence analysis and promoter cloning

Global pair-wise alignment of three kb upstream and two kb downstream of the predicted human and mouse transcription start sites were done using mVISTA-AVID (Frazer et al. 2004), using default parameters, to identify putative conserved regulatory sequences. Predicted LEF1/TCF binding sites (described by matrix family V$LEFF) were identified using MatInspector (Genomatix) with the default (optimized) threshold. Gene annotation information is derived from the Ensembl database. The promoter sequences after PCR amplification (primers sequences below) were attached to the firefly luciferase gene of the pTA-Luc plasmid (Promega), which lacks the minimal TA promoter, as described previously (Wisniewska et al. 2010).

Etv1 (ENSMUST00000095767) -2196, +279

(forward with MluI site Etv1_M_F TATCTTACGCGTAGACAAAGGGTCCTTAGTC

reverse with XhoI site Etv1_X_R ATCTAACTCGAGAATTATTGCCCAGCACTTCC)

Foxp2 (ENSMUST00000031545) -1892, +93

(F with KpnI site ACACCGGTACCGGAGACATGTGGAG

Reverse with MluI site CGAACGCGTCCCACACTGATGGC)

Gbx2 (ENSMUST00000036954) 0 acc to NM_010262.3, -1964, +176

(forward with KpnI site, ATCTTAGGTACCAAGGTAGTGGCATTGAAG

reverse with MluI site, TATCTTACGCGTTCGCCGGAACCGCTC)

Mef2c 0 acc to ENST00000437473 -1394, +178

(forward with XhoI site: 5’-ATCTAACTCGAGCATTCTCCGAGGAAGCTGTC-3’

reverse with XhoI site: 5’-ATCTAACTCGAGTTTGTCCAGCCTTGAAGTGC-3’

Nr4a2 Nurr1 (ENSMUST00000028166) -1735, +299

forward with KpnI site, ATCTTAGGTACCTTTCGTTCGCGTGGAATCG

reverse with MluI site, TATCTTACGCGTTCAACTCCGCCGAAGTGC

Pou4f1 Brn3a (ENSMUST00000053016) -1888, +12

forward with MluI site, TATCTTACGCGTTGGACTCACTCTTTCCCTC

reverse with XhoI site, ATCTAACTCGAGATCCCGCTTCTCCGAGAG

Rora (ENSMUST00000034766) -1905, +36

forward with XhoI site, ATCTAACTCGAGACTTCTGTCCCAGAGGAAG

reverse with XhoI site, ATCTAACTCGAGCCGGAGCTGACTCCATG

Zfp804a (ENSMUST00000047527) -1470, +123

forward with MluI site, TTACTTACGCGTATTTCTGGGTGACCTCCTC

reverse with XhoI site, ATCTAACTCGAGTTGCTGGTCCATCGCACTG

Zic1 (ENSMUST00000034927) -2133, -9

Forward mZic1 MluI, ATGTGAAAGGACGCGTTGAAGG

Reverse mZic NheI, ATAGCAGCTAGCATTCCTGCAG

Plasmids

Constructs used for transfection were: CMV expression plasmids: mouse *Lef1* (from Prof. Rudolf Grosschedl, Max Planck Institute of Immunobiology, Freiburg, Germany), mouse Tcf7l2-E2, Tcf7l2-S3 (Nagalski et al. 2013), mouse β-catenin (from Prof. Rolf Kemler, Max

Planck Institute of Immunobiology). Deletion constructs of Tcf7l2-S3 isoforms (i.e.: $_{\Delta 30}$TCF7L2-S, and $_{\Delta 161}$TCF7L2-S) were PCR amplified from the Tcf7l2-S3 plasmid and cloned to the pCG vector.

Luciferase Assay

HeLa cells (ATTC) were cultured in Dulbecco's modified Eagle's medium (Invitrogen) containing 10% fetal bovine serum (Sigma) and 100 units/ml penicillin-streptomycin. 2 × 104 cells (per well of a 24-well plate) were transfected using polyethylene-amine reagent, 24 h after plating. The cells received a mixture of 250 ng of reporter plasmids with promoters, 250 ng of expression vectors, and reporter vector pRL-TK expressing luciferase from Renilla (Promega). The total amount of transfected DNA was kept constant by adding appropriate amounts of the empty expression vector pCG. Two days after transfection, the cells were washed with PBS, lysed in Passive Lysis Buffer (Promega), and the obtained signal was detected with a Lumistar Galaxy (BMG) luminometer, using jointly the luciferase buffer (50mM Tris-HCl, 11mM MgCl2, 10mM dithiotreitol, 0.2mM ATP, 0.5mM D-Luciferin, 0.25mM Coenzyme A) and the Renilla luciferase buffer (50mM Tris-HCl, 100mM NaCl, 2,5μM coelenterazine). Reporter gene activities were normalized against Renilla luciferase activity. Difference to the relative mean for each reporter was calculated with two-tailed Mann-Whitney test.

Supplementary Literature

Frazer KA, Pachter L, Poliakov A, Rubin EM, Dubchak I. 2004. VISTA: computational tools for comparative genomics. Nucleic Acids Res 32:W273-279.
